# Distinct abdominal and gluteal adipose tissue transcriptome signatures are altered by exercise training in African women with obesity

**DOI:** 10.1038/s41598-020-66868-z

**Published:** 2020-06-24

**Authors:** Pamela A. Nono Nankam, Matthias Blüher, Stephanie Kehr, Nora Klöting, Knut Krohn, Kevin Adams, Peter F. Stadler, Amy E. Mendham, Julia H. Goedecke

**Affiliations:** 1 Division of Exercise Science and Sports Medicine, Department of Human Biology, University of Cape Town, Cape Town, South Africa; 2 Department of Endocrinology, Faculty of Medicine, University of Leipzig, Leipzig, Germany; 3 Helmholtz Institute for Metabolic, Obesity and Vascular Research (HI-MAG) of the Helmholtz Zentrum München at the University of Leipzig and University Hospital Leipzig, Leipzig, Germany; 4 Bioinformatics Group, Department of Computer Science, and Interdisciplinary Center for Bioinformatics, University of Leipzig, Leipzig, Germany; 5 Core Unit DNA-Technologies, Medical Faculty, University Leipzig, Leipzig, Germany; 6 Non-communicable Diseases Research Unit, South African Medical Research Council, Tygerberg, Cape Town South Africa

**Keywords:** Gene expression, Obesity

## Abstract

The differential associations of adipose depots with metabolic risk during obesity have been proposed to be controlled by environmental and genetic factors. We evaluated the regional differences in transcriptome signatures between abdominal (aSAT) and gluteal subcutaneous adipose tissue (gSAT) in obese black South African women and tested the hypothesis that 12-week exercise training alters gene expression patterns in a depot-specific manner. Twelve young women performed 12-weeks of supervised aerobic and resistance training. Pre- and post-intervention measurements included peak oxygen consumption (VO_2peak_), whole-body composition and unbiased gene expression analysis of SAT depots. VO_2peak_ increased, body weight decreased, and body fat distribution improved with exercise training (p < 0.05). The expression of 15 genes, mainly associated with embryonic development, differed between SAT depots at baseline, whereas 318 genes were differentially expressed post-training (p < 0.05). Four developmental genes were differentially expressed between these depots at both time points (*HOXA5, DMRT2*, *DMRT3* and *CSN1S1*). Exercise training induced changes in the expression of genes associated with immune and inflammatory responses, and lipid metabolism in gSAT, and muscle-associated processes in aSAT. This study showed differences in developmental processes regulating SAT distribution and expandability of distinct depots, and depot-specific adaptation to exercise training in black South African women with obesity.

## Introduction

Body fat distribution is an independent contributor to the development of obesity-associated metabolic diseases^1,2^. While central fat accumulation (both in visceral and abdominal subcutaneous areas) has been associated with greater risk for developing metabolic diseases, lower-body fat (gluteo-femoral) appears to have positive effects on metabolic health^3–5^. The mechanisms underlying these depot-specific associations with metabolic risks are not fully understood. However, environmental and genetic factors could mediate differences in gene expression, metabolism and function between fat depots^6,7^.

Adipose tissue (AT) accumulation follows a specific individual pattern during positive energy balance, with the preferential expansion of subcutaneous vs. visceral depot, largely determined by heritable factors^1^. This specific response to excess calories could also be influenced by site-specific sets of developmental genes^8,9^. Numerous studies have reported an extensive number of differentially expressed genes (DEGs) between visceral adipose tissue (VAT) and subcutaneous adipose tissue (SAT) (both abdominal and gluteal)^4,8,10,11^. This suggests different developmental lineage, potentially explaining depot-specific adipose metabolism and association with metabolic risk. However, whether the difference in embryonic origin exists within SAT depots remains elusive. Indeed, in addition to their functional differences and their distinctive associations with metabolic risk, epigenetic differences between abdominal SAT (aSAT) and gluteal SAT (gSAT) have been shown^5,8,12,13^. Notably, these findings were mainly derived from studies in European populations and no data is available on the depot-specific SAT transcriptome profile in an exclusively African population, highlighting the need to study SAT signatures in Africans.

Sub-Saharan Africa is experiencing exponential growth in the prevalence of obesity with more women (15%) affected than men (6%)^14^. South Africa presents with the highest prevalence of overweight and obesity in women (40%) in this region^15^, along with a high incidence of type 2 diabetes (14.7%)^16^. Moreover, despite African women having more gluteo-femoral fat, and relatively less VAT^17,18^, they are more insulin resistant than their white (European ancestry) counterparts for the same level of body mass index (BMI)^19^. Furthermore, gSAT in black African women is characterized by higher expression of genes linked to hypoxia, fibrosis and inflammation than that of their white counterparts^20^. This suggests that the gene expression profile of SAT depots is ethnic-specific and consequently, might have divergent metabolic effects in black African compared to European women.

Excessive fat accumulation consecutive to chronic positive energy balance is sustained by low physical activity^21^. Exercise training (one of the principal components of physical activity) can exert beneficial effects on metabolic health via modifications in AT metabolism^22^. Specifically, previous studies have shown changes in AT lipid metabolism (lipolysis, lipogenesis), fatty acid uptake and oxidation, as well as glucose homeostasis and mitochondrial activity after exercise training in individuals with obesity^23,24^. However, the extent of these changes on SAT transcriptome has not yet been investigated in African populations.

Using an unbiased microarray analysis approach, this study aimed to evaluate the regional differences in gene expression profiles between aSAT and gSAT in obese black South African women and tested the hypothesis that the gene expression pattern is altered in a depot-specific manner in response to 12-weeks of combined aerobic and resistance exercise training.

## Results

### Participants’ characteristics

The basic characteristics of the participants involved in this study are presented in Table 1. These premenopausal women were on average 23 ± 3 years of age with a BMI of 33.8 ± 2.6 kg/m². They were centrally obese, with waist circumference (WC) of 102 (±5) cm and significantly more subcutaneous (5451 ± 803 cm^3^) than visceral fat (971 ± 330 cm^3^) (Table 1). In response to exercise training, cardiorespiratory fitness (VO_2peak_) increased (p < 0.05), and weight, BMI, waist circumference, waist/hip ratio (WHR) decreased. While fat-free soft tissue mass (FFSTM) and total body fat (%) did not change, both android and gynoid fat mass (%) decreased (p < 0.05), and there was a tendency for VAT and SAT volumes to decrease (p < 0.1). There was no significant difference in daily energy or macronutrient intake after the 12-week exercise training intervention (Supplementary Table S1).

**Table 1:** Participant’s characteristics before and after exercise training.

Variables	Pre (n = 12)	Post (n = 12)	P Value
VO_2peak_ (ml/min)	2121 ± 217	2245 ± 204	**0.023**
VO_2peak_ (ml/min/kg FFSTM)	58.9 ± 5.0	62.4 ± 4.6	**0.007**
VO_2peak_ (ml/kg/min)	25.9 ± 2.2	28.0 ± 2.6	**0.008**
Weight (kg)	82.2 ± 7.2	80.5 ± 7.5	**0.004**
BMI (kg/m²)	33.8 ± 2.6	33.1 ± 2.8	**0.004**
WC (cm)	102.0 ± 5.5	97.7 ± 5.42	**0.002**
WHR	0.90 ± 0.09	0.88 ± 0.06	**0.019**
FFSTM (kg)	36.1 ± 3.3	36.1 ± 3.4	0.943
Body FM (%)	49.7 ± 1.6	49.1 ± 1.5	0.205
Android FM (%)	8.5 ± 1.1	8.2 ± 1.2	**0.010**
Gynoid FM (%)	18.3 ± 1.9	18.0 ± 1.8	**0.033**
VAT (cm^3^)	971 ± 330	909 ± 384	0.086
SAT (cm^3^)	5451 ± 803	5266 ± 944	0.062

Data presented as means ± SD; p values represent the differences between baseline and post-training. VO2peak, peak oxygen consumption, used as a measure of cardiorespiratory fitness; BMI, body mass index; WC, waist circumference; WHR, waist and hip ratio; FM, Fat-mass; FFSTM, fat-free soft tissue mass; VAT, Visceral adipose tissue; SAT, Subcutaneous adipose tissue.

### Gene expression differences between SAT depots at baseline

**Figure 1:**
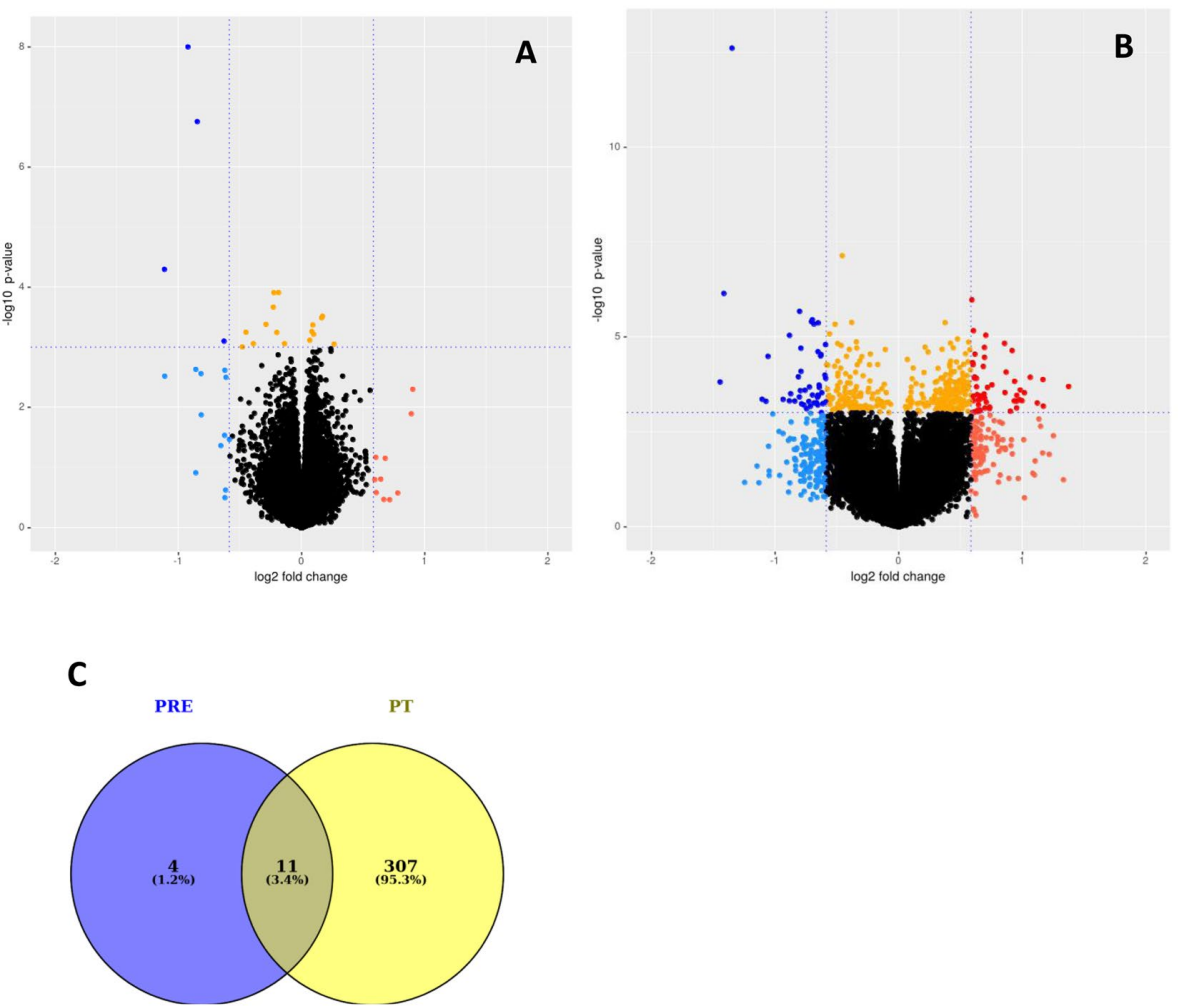
Comparison of abdominal SAT vs gluteal SAT, before **(A)** and after **(B)** exercise training respectively. Blue: Higher gene expression in aSAT (LFC < −0.58. Red: Lower gene expression in aSAT (LFC > 0.58), Orange: p-value < 0.001. The horizontal line corresponds to p < 0.001 and the vertical lines mark a |LFC | > 0.58. **(A)** aSAT vs gSAT at baseline. **(B)** aSAT vs gSAT after exercise training. **(C)** Overlap of genes with changed expression (|LFC | > 0.58) between gSAT and aSAT. PRE: difference between aSAT and gSAT pre-exercise training (at baseline); PT: difference between aSAT and gSAT post-exercise training.

The differences in gene expression profiles between aSAT and gSAT at baseline and after exercise training are presented in Fig. 1A,B, respectively, and the multidimensional scaling (MDS) plots are shown in Supplementary Figure 3. At baseline, only 15 genes were differentially expressed between aSAT and gSAT (|log2 fold change (LFC) | ≥ 0.58; p ≤ 0.05) with the expression of 13 genes being higher and 2 genes being lower in aSAT vs. gSAT (Fig. 1A; Table 2). Given this small number of DEGs, the motif enrichment analysis was not reliable, preventing us from reporting on putative transcription factor binding sites (TFBS) at baseline (see supplementary file).

**Table 2:** *List of all differentially expressed genes between SAT depots before exercise training based on log2 fold change* > 0.58 (up and down-regulated).

SYMBOL	SEARCH_KEY	logFC	DEFINITION
***Higher in aSAT than gSAT***			
**DMRT2**	**NM_181872.1**	−**1.11**	**Homo sapiens doublesex and mab-3 related transcription factor 2 (DMRT2), transcript variant 1, mRNA**.
**HOXA5**	**NM_019102.2**	−**1.11**	**Homo sapiens homeobox A5 (HOXA5), mRNA**.
**DMRT3**	**NM_021240.2**	−**0.92**	**Homo sapiens doublesex and mab-3 related transcription factor 3 (DMRT3), mRNA**.
RSPO3	NM_032784.3	−0.86	Homo sapiens R-spondin 3 homolog (Xenopus laevis) (RSPO3), mRNA.
IRX2	NM_033267.2	−0.85	Homo sapiens iroquois homeobox 2 (IRX2), mRNA.
C6	NM_000065.1	−0.82	Homo sapiens complement component 6 (C6), mRNA.
ALDH1A1	NM_000689.3	−0.81	Homo sapiens aldehyde dehydrogenase 1 family, member A1 (ALDH1A1), mRNA.
ALDH1A1	NM_000689.3	−0.66	Homo sapiens aldehyde dehydrogenase 1 family, member A1 (ALDH1A1), mRNA.
HOXB8	NM_024016.2	−0.63	Homo sapiens homeobox B8 (HOXB8), mRNA.
FGFBP2	NM_031950.2	−0.62	Homo sapiens fibroblast growth factor binding protein 2 (FGFBP2), mRNA.
IRX5	NM_005853.4	−0.62	Homo sapiens iroquois homeobox protein 5 (IRX5), mRNA.
LOC440928	XM_942885.1	−0.61	PREDICTED: Homo sapiens hypothetical LOC440928 (LOC440928), mRNA.
PPP1R1B	NM_181505.1	−0.59	Homo sapiens protein phosphatase 1, regulatory (inhibitor) subunit 1B (dopamine and cAMP regulated phosphoprotein, DARPP-32) (PPP1R1B), transcript variant 2, mRNA.
***Lower in aSAT than gSAT***			
**CSN1S1**	**NM_001890.1**	**0.90**	**Homo sapiens casein alpha s1 (CSN1S1), transcript variant 1, mRNA**.
**CSN1S1**	**NM_001025104.1**	**0.89**	**Homo sapiens casein alpha s1 (CSN1S1), transcript variant 1, mRNA**.

### Gene expression differences between SAT depots after 12-week exercise training

**Figure 2:**
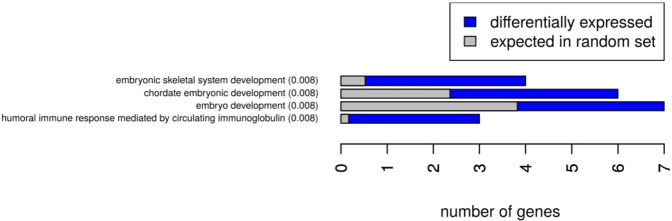
GO term enrichment of differentially expressed genes between gluteal and abdominal SAT at baseline.

In contrast to the small number of DEGs between fat depots at baseline, we identified 318 DEGs after exercise training (Fig. 1B); with the expression of 166 genes being higher and 152 genes being lower in aSAT vs gSAT (Table 3 for the top 10 DEGs; Supplementary Table S2). Notably, 11 of these genes overlapped between the SAT depots before and after exercise training (Fig. 1C). The most prominent genes distinguishing the SAT depots at both time points (highlighted in bold in Tables 2 and 3) were *doublesex and mab-3 related transcription factor (DMRT)2*, *DMRT3*, and *homeobox (HOX) A5*, with higher expression levels in aSAT compared to gSAT; as well as *casein alpha s1 (CSN1S1)*, with lower expression in aSAT vs gSAT. Several HOX genes were found with higher expression levels in aSAT than gSAT at baseline or after exercise training (*HOXA3*, *HOXA5*, *HOXA9*, *HOXB5*, *HOXB8*, *HOPX*, iroquois homeobox *(IRX) 2* and *IRX5*). The evaluation of the biological processes represented by the DEGs between the depots at both time points using STRING showed that they were mainly enriched for gene ontology (GO) terms: embryonic development, anatomical structure and developmental processes at baseline (Fig. 2), and more diverse functional-related processes at post-training (Supplementary Fig. 1). In addition, motif enrichment analyses using hypergeometric optimization of motif enrichment (HOMER) were completed and a detailed report is provided in the supplementary document. Briefly, we identified known and/or *de novo* enriched motifs for higher and lower DEGs in aSAT vs gSAT. In the higher DEGs in aSAT vs. gSAT, the predicted enriched motifs were NF1 half-site and LRF binding motif (associated with adipogenesis and glycolysis), with no enriched *de novo* motifs. In contrast, in the lower DEGs in aSAT vs gSAT we identified an enrichment of RUNX binding site (associated with the immune system and pro-inflammation; see supplementary document), USF1 binding site, and E-box promoter element (which can act as a USF1 binding site^25^. The transcription factor USF1 is linked to familial combined hyperlipidemia and has been associated with the metabolism of brown adipose tissue in mice^26^. Furthermore, we identified *de novo* enriched motifs for lower DEGs in aSAT vs gSAT involved in adipocyte differentiation (ZNF467)^27^ and to immune system response in humans (PRDM1)^28^.

**Table 3:** *Top 10 differentially expressed genes between SAT depots after exercise training based on log2 fold change* > 0.58 (up and down-regulated).

SYMBOL	SEARCH_KEY	logFC	DEFINITION
***Higher in aSAT than gSAT***			
**HOXA5**	**NM_019102.2**	**−1.44**	**Homo sapiens homeobox A5 (HOXA5), mRNA**.
**DMRT2**	**NM_181872.1**	−**1.41**	**Homo sapiens doublesex and mab-3 related transcription factor 2 (DMRT2), transcript variant 1, mRNA**.
**DMRT3**	**NM_021240.2**	−**1.35**	**Homo sapiens doublesex and mab-3 related transcription factor 3 (DMRT3), mRNA**.
MYL2	NM_000432.2	−1.15	Homo sapiens myosin, light chain 2, regulatory, cardiac, slow (MYL2), mRNA.
CDKN1B	NM_004064.2	−1.10	Homo sapiens cyclin-dependent kinase inhibitor 1B (p27, Kip1) (CDKN1B), mRNA.
MYH11	NM_002474.1	−1.07	Homo sapiens myosin, heavy chain 11, smooth muscle (MYH11), transcript variant SM1A, mRNA.
ANGPT2	NM_001118888.1	−1.06	Homo sapiens angiopoietin 2 (ANGPT2), transcript variant 3, mRNA.
LOC649841	XM_938906.1	−1.05	PREDICTED: Homo sapiens similar to protein immuno-reactive with anti-PTH polyclonal antibodies (LOC649841), mRNA.
PARM1	NM_015393.2	−1.05	Homo sapiens prostate androgen-regulated mucin-like protein 1 (PARM1), mRNA.
MYH7	NM_000257.1	−1.05	Homo sapiens myosin, heavy chain 7, cardiac muscle, beta (MYH7), mRNA.
***Lower in aSAT than gSAT***			
**CSN1S1**	**NM_001025104.1**	**1.37**	**Homo sapiens casein alpha s1 (CSN1S1), transcript variant 1, mRNA**.
SPP1	NM_001040058.1	1.25	Homo sapiens secreted phosphoprotein 1 (SPP1), transcript variant 1, mRNA.
LOC644936	NR_004845.1	1.22	Homo sapiens cytoplasmic beta-actin pseudogene (LOC644936), non-coding RNA.
IFI30	NM_006332.3	1.17	Homo sapiens interferon, gamma-inducible protein 30 (IFI30), mRNA.
LAPTM5	NM_006762.1	1.17	Homo sapiens lysosomal multispanning membrane protein 5 (LAPTM5), mRNA.
SPP1	NM_000582.2	1.16	Homo sapiens secreted phosphoprotein 1 (SPP1), transcript variant 2, mRNA.
FCGBP	NM_003890.1	1.15	Homo sapiens Fc fragment of IgG binding protein (FCGBP), mRNA.
TM4SF19	NM_138461.1	1.13	PREDICTED: Homo sapiens transmembrane 4 L six family member 19, transcript variant 2 (TM4SF19), mRNA.
CSN1S1	NM_001890.1	1.12	Homo sapiens casein alpha s1 (CSN1S1), transcript variant 1, mRNA.
FOSB	NM_006732.1	1.10	Homo sapiens FBJ murine osteosarcoma viral oncogene homolog B (FOSB), mRNA.

### Gene expression in gluteal SAT in response to exercise training

**Figure 3:**
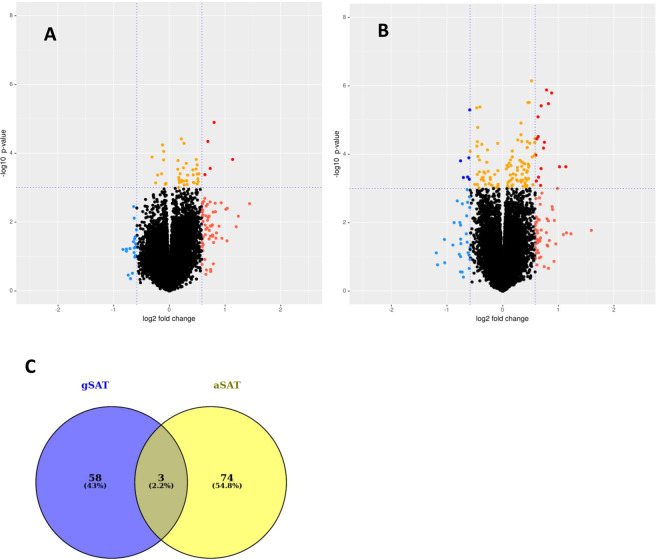
Comparison of gene expression changes induced by exercise training in gSAT **(A)** and aSAT **(B)**, respectively. The volcano plots highlight genes with p-value < 0.001 and log2 fold change |LFC | > 0.58. Red: gene expression upregulated after exercise training (LFC > 0.58), Blue: gene expression downregulated after exercise training (LFC < −0.58). Orange: p-value < 0.001. The horizontal line corresponds to p < 0.001 and the vertical lines mark a | *LFC* | > *0.58*. **(A)** Gene expression changes in gSAT after exercise. **(B)** Gene expression changes in aSAT after exercise training. **(C)** Overlapping genes with changed expression* (|LFC*| > 0.58) in both depots before and after exercise training.

**Figure 4:**
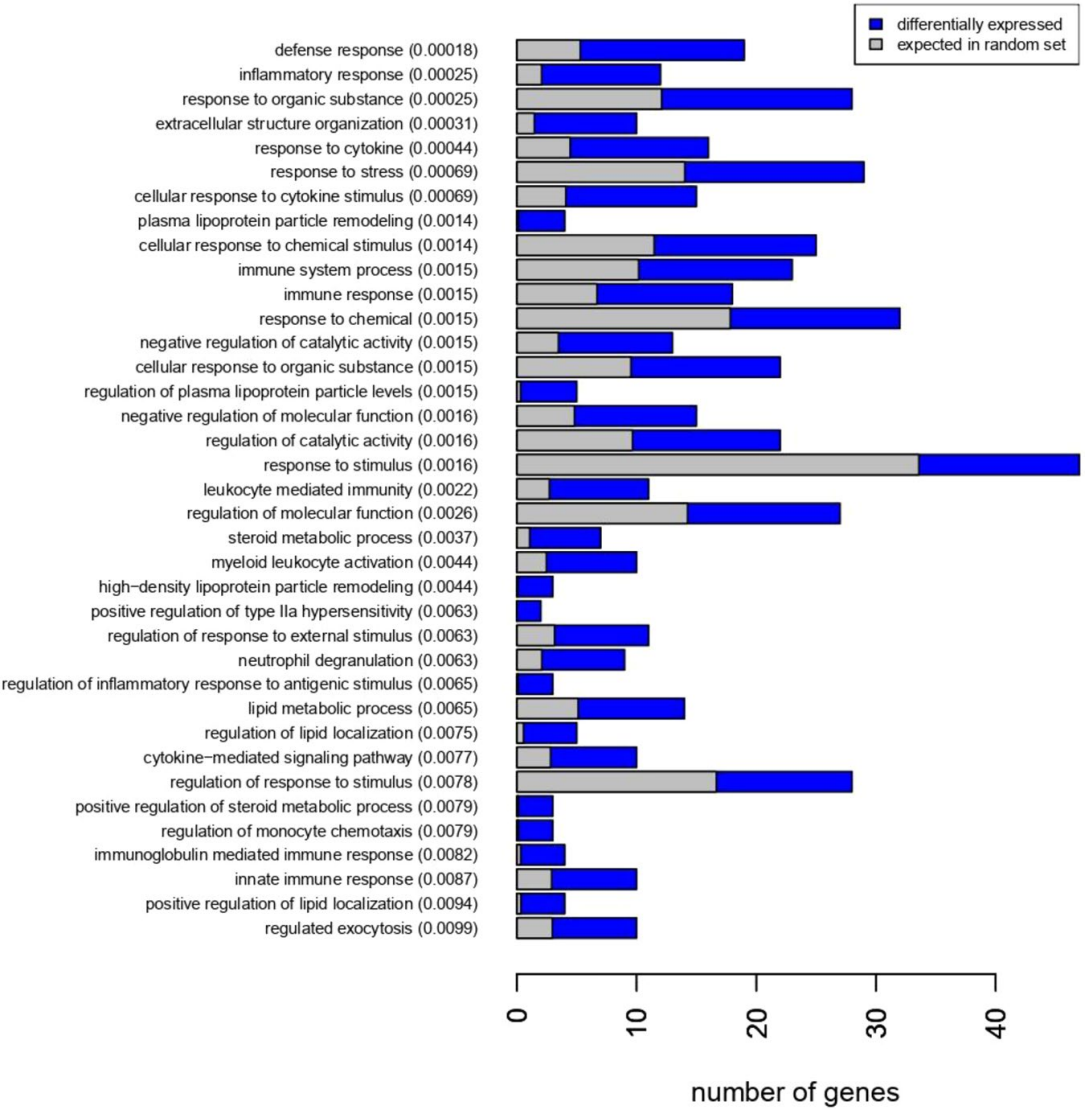
GO-term Enrichment of differentially expressed genes in gluteal SAT after exercise training.

To evaluate the depot-specific response to exercise training, we compared the gene expression level in gSAT (Fig. 3A) and aSAT (Fig. 3B) pre and post-training, respectively. In gSAT, we identified 61 DEGs (|LFC | ≥ 0.58; p ≤ 0.05) with 54 genes upregulated and 7 genes downregulated in response to exercise training (Table 4; Supplementary Table S3). The biological processes most affected by exercise training (Fig. 4) were immune and inflammatory responses (e.g. *SPP1*, *CCL22*, *MMP9, CHI3L1, HP*), regulation of lipid metabolism (e.g. *APOE, PLTP, SPP1, C3, APOC1, SREBF1*) and regulation of plasma lipoprotein particle levels and remodelling (e.g. *APOC1, APOE, PLA2G7, PLTP, LIPA*). Moreover, the HOMER analysis on the combined set of up- and down-regulated genes (due to the low number of downregulated genes) showed enrichment for the transcription factor complex AP-1 similar to binding motifs of Fos-related factors or Jun family. Additionally, two known predicted binding motifs: activation transcription factor 3 (Atf3) and basic leucine zipper ATF-like transcription factor (BATF) were identified (see supplementary document). Atf3 belongs to the cAMP-responsive element-binding (CREB) class and interacts with Jun-related factors, and BATF mediates the dimerization of Fos and Jun factors to form the AP-1 transcription factor complex. The AP-1 transcription factor complex is a regulator of cell proliferation, differentiation and transformation in response to stimuli like cytokines, stress or growth factor^29^.

**Table 4:** *Differentially expressed genes in gluteal SAT in response to exercise training based on log2 fold change* > 0.58 (Top 10 up-regulated genes and all down-regulated genes).

SYMBOL	SEARCH_KEY	logFC	DEFINITION
***Up-regulated genes in gSAT after exercise training***			
MMP9	NM_004994.2	−1.44	Homo sapiens matrix metallopeptidase 9 (gelatinase B, 92 kDa gelatinase, 92 kDa type IV collagenase) (MMP9), mRNA.
SPP1	NM_001040058.1	−1.24	Homo sapiens secreted phosphoprotein 1 (SPP1), transcript variant 1, mRNA.
SPP1	NM_000582.2	−1.20	Homo sapiens secreted phosphoprotein 1 (SPP1), transcript variant 2, mRNA.
APOC1	NM_001645.3	−1.14	Homo sapiens apolipoprotein C-I (APOC1), mRNA.
ITGAX	NM_000887.3	−1.04	Homo sapiens integrin, alpha X (complement component 3 receptor 4 subunit) (ITGAX), mRNA.
TM4SF19	NM_138461.2	−1.04	Homo sapiens transmembrane 4 L six family member 19 (TM4SF19), mRNA.
IFI30	NM_006332.3	−1.02	Homo sapiens interferon, gamma-inducible protein 30 (IFI30), mRNA.
PLA2G7	NM_005084.2	−0.94	Homo sapiens phospholipase A2, group VII (platelet-activating factor acetylhydrolase, plasma) (PLA2G7), mRNA.
LAPTM5	NM_006762.1	−0.93	Homo sapiens lysosomal multispanning membrane protein 5 (LAPTM5), mRNA.
CISH	NM_145071.1	−0.89	Homo sapiens cytokine inducible SH2-containing protein (CISH), mRNA.
***Down-regulated genes in gSAT after exercise training***			
LOC651309	XM_942586.1	0.64	PREDICTED: Homo sapiens hypothetical protein LOC651309 (LOC651309), mRNA.
PCDH9	NM_020403.3	0.64	Homo sapiens protocadherin 9 (PCDH9), transcript variant 1, mRNA.
NTM	NM_016522.2	0.63	Homo sapiens neurotrimin (NTM), transcript variant 2, mRNA.
SLIT2	NM_004787.1	0.62	Homo sapiens slit homolog 2 (Drosophila) (SLIT2), mRNA.
NUTF2	NM_005796.1	0.61	Homo sapiens nuclear transport factor 2 (NUTF2), mRNA.
	Hs.99472	0.60	Homo sapiens mRNA; cDNA DKFZp564O0862 (from clone DKFZp564O0862)
FAM13A	NM_014883.2	0.59	Homo sapiens family with sequence similarity 13, member A (FAM13A), transcript variant 1, mRNA.

### Gene expression in abdominal SAT in response to exercise training

**Figure 5:**
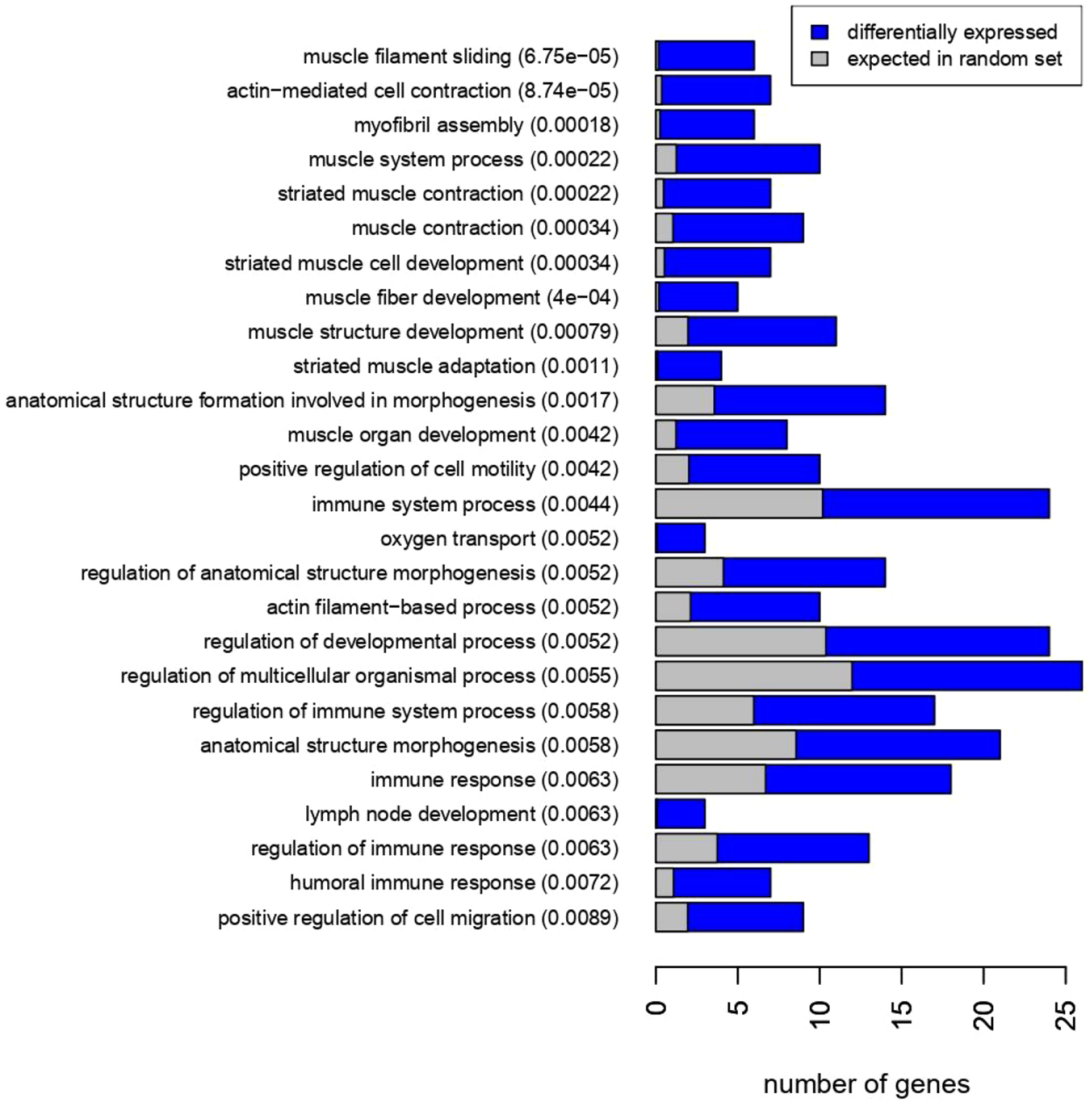
GO-term Enrichment of differentially expressed genes in abdominal SAT after exercise training.

Within aSAT, the expression of 77 genes changed in response to exercise training (|LFC | ≥ 0.58; p ≤ 0.05), with 55 genes upregulated and 22 genes downregulated (Table 5; Supplementary Table S4). Surprisingly, these genes mostly represented muscle-associated processes (e.g. *ACTA1, MYH7, MYL2, TCAP, CKM*) and immune response (e.g. *HP, COL1A1, CD3D, IL7R*) (Fig. 5). Notably, only 3 genes had commonly altered expression levels in aSAT and gSAT after exercise training (*COL1A1, HP, CILP*) (Fig. 3C). We subsequently combined the up- and down-regulated genes in aSAT in response to exercise training to identify enriched motifs, as the number of downregulated genes was only 22. Four known TFBS were found in the promoter regions of the DEGs (CarG-box DNA motif, Mef2b RUNX1 and Gfi1b). Further, Smad2 was the best match of the predicted *de novo* motifs, which is a regulator of multiple cellular processes, such as cell proliferation, apoptosis and differentiation (see supplementary document).

**Table 5:** *Top 10 differentially expressed genes in abdominal SAT in response to exercise training based on log2 fold change* > 0.58 (up and down-regulated).

SYMBOL	SEARCH_KEY	logFC	DEFINITION
***Up-regulated genes in aSAT after exercise training***			
ACTA1	NM_001100.3	−1.59	Homo sapiens actin, alpha 1, skeletal muscle (ACTA1), mRNA.
FOLR3	NM_000804.2	−1.23	Homo sapiens folate receptor 3 (gamma) (FOLR3), mRNA.
MYL2	NM_000432.2	−1.15	Homo sapiens myosin, light chain 2, regulatory, cardiac, slow (MYL2), mRNA.
CHI3L2	NM_004000.2	−1.13	Homo sapiens chitinase 3-like 2 (CHI3L2), transcript variant 1, mRNA.
MYH7	NM_000257.1	−1.09	Homo sapiens myosin, heavy chain 7, cardiac muscle, beta (MYH7), mRNA.
COL1A1	NM_000088.2	−1.02	Homo sapiens collagen, type I, alpha 1 (COL1A1), mRNA.
FLNC	NM_001458.2	−0.98	Homo sapiens filamin C, gamma (actin binding protein 280) (FLNC), mRNA.
CKM	NM_001824.2	−0.96	Homo sapiens creatine kinase, muscle (CKM), mRNA.
LTB	NM_002341.1	−0.93	Homo sapiens lymphotoxin beta (TNF superfamily, member 3) (LTB), transcript variant 1, mRNA.
FNDC1	NM_032532.1	−0.90	Homo sapiens fibronectin type III domain containing 1 (FNDC1), mRNA.
***Down-regulated genes in aSAT after exercise training***			
FOS	NM_005252.2	1.05	Homo sapiens v-fos FBJ murine osteosarcoma viral oncogene homolog (FOS), mRNA.
FOSB	NM_006732.1	0.89	Homo sapiens FBJ murine osteosarcoma viral oncogene homolog B (FOSB), mRNA.
CSN1S1	NM_001025104.1	0.87	Homo sapiens casein alpha s1 (CSN1S1), transcript variant 1, mRNA.
MYOC	NM_000261.1	0.82	Homo sapiens myocilin, trabecular meshwork inducible glucocorticoid response (MYOC), mRNA.
TWIST1	NM_000474.3	0.77	Homo sapiens twist homolog 1 (Drosophila) (TWIST1), mRNA.
LOC643911	XR_042101.1	0.76	PREDICTED: Homo sapiens hCG1815491 (LOC643911), miscRNA.
CSN1S1	NM_001890.1	0.76	Homo sapiens casein alpha s1 (CSN1S1), transcript variant 1, mRNA.
MYOC	NM_000261.1	0.75	Homo sapiens myocilin, trabecular meshwork inducible glucocorticoid response (MYOC), mRNA.
THBS4	NM_003248.3	0.75	Homo sapiens thrombospondin 4 (THBS4), mRNA.
LOC643911	XM_931911.1	0.72	PREDICTED: Homo sapiens hypothetical LOC643911 (LOC643911), mRNA.

### Differential mRNA expression in adipocytes and cells of the stromal vascular fraction

To assess the cellular origin of the whole-SAT mRNA expression of *HOXA5, DMRT2, DMRT3, R-Spondin 3 (RSPO3), CS1N1* and *matrix metallopeptidase 9 (MMP9)*, mRNA levels of these genes were measured in adipocytes and the stromal vascular fraction (SVF) of an independent cohort (n = 29) detailed in the Method section. While *RSPO3* and *CS1N1* mRNA levels were higher in adipocytes compared to SVF (p < 0.001), *MMP9* mRNA level was higher in SVF than in adipocytes (p < 0.0001) (Supplementary Fig. 2). In addition, there was a trend towards a higher expression of *DMRT3* (p = 0.065) and *HOXA5* (p = 0.075) in adipocytes compared to SVF, but *DMRT2* expression was not different between these cell fractions (p = 0.178) (Supplementary Fig. 2).

## Discussion

This is the first study to our knowledge that has investigated the regional differences in SAT transcriptome signatures in obese African women and showed differences in gene expression profiles between aSAT and gSAT at baseline and in response to exercise training. As a key result of our unbiased analysis, we found 15 DEGs between these fat depots at baseline, which were mainly associated with embryonic development (e.g. *HOXA5, DMRT2, HOXB8, IRX5, IRX2*) and regulation of anatomical structure morphogenesis (e.g. *DMRT2, DMRT3, HOXA5, RSPO3*). Depot-specific AT transcriptome signatures were strongly pronounced in response to the 12-weeks structured exercise training program, such that 318 genes were differentially expressed between the depots, and only three genes commonly changed in aSAT and gSAT. Interestingly, we identified four developmental genes as the most prominent DEGs between SAT depots (*DMRT2*, *DMRT3* and *HOXA5* higher, and *CSN1S1* lower expressed in aSAT compared to gSAT) that were unaffected by exercise training.

Adipose tissue depots have been proposed to arise from different mesodermal layers during embryonic development, directed through epigenetic-programmed mechanisms^8,30^. *HOX* family is among the most studied families of developmental genes in AT and has been widely identified as fundamental in controlling the body plan along the anterior-posterior axis in diverse species including humans^8^. *HOX* genes have also been suggested as determinants of early regional differentiation by directing the evolution and differentiation from mesoderm layers to different AT depots^12^. We have previously shown in both mice and humans (Europeans ancestry) that multiple *HOX* genes are involved in embryonic development and pattern specification (e.g. *HOXA5, HOXC8, HOXC9*), and play an important role in obesity and body fat distribution, with potential functional differences between distinct AT depots^9^. However, to our knowledge, no studies have explored HOX genes expression in an African population that may explain their distinct body fat distribution. Moreover, there is a lack of data on the depot-specific SAT transcriptome profile in an exclusively African population, highlighting the need to study SAT signatures in Africans and the novelty of the present study. We show that the following *HOX* genes were more highly expressed in aSAT than in gSAT: *HOXA5* (at both time points), *IRX2*, *HOXB8 and IRX5* (at baseline), and *IRX2*, *HOPX*, *HOXA3*, *HOXB5* and *HOXA9* (after exercise training). Higher *HOXA5* expression in aSAT compared to gSAT has been previously shown in obese men and women of European ancestry, as well as higher expression of this gene and other HOX genes in the SVF of aSAT compared to that of gSAT^12^. Furthermore, high levels of *HOXA5* have been closely related to BMI and body fat distribution in both obese humans and animal models^6,9,12,31,32^. In addition, several homeobox genes were showed to be upregulated in aSAT and VAT (e.g. *HOXA3*, *HOXA9, HOXB5, HOXB8, IRX5, IRX2* and *HOPX*)^5,7,9,12,13,33^. This suggests a similar developmental origin of aSAT and VAT, rather than aSAT and gSAT^8^. Notably, *IRX* genes are potentially involved in the mechanisms underlying the genetic association between *FTO* and obesity^34^. The causal variant (*rs1421085*) of this association eliminates *IRX3* and *IRX5* repression, leading to a cell-autonomous shift from browning and thermogenesis of white adipocytes to lipid storage, increased fat stores and body-weight gain^34^. Higher expression of *IRX* genes in aSAT could therefore be one of the causal factors in the detrimental central fat accumulation (aSAT) in obese individuals. However, this should be further investigated in a cohort of African women characterized by low central and high gynoid fat accumulation.

Unlike the homeobox family, *DMRT* family has been less studied in the context of obesity and body fat distribution. Mostly involved in sex differentiation and gonadal development during embryogenesis in a wide range of species^35–37^, the expression of *DMRT* genes has also been reported in aSAT of individuals with obesity^37^. In the present study, we found higher expression of *DMRT2* and *DMRT3* in aSAT vs gSAT at baseline and post-exercise training, similar to findings from Passaro *et al*. in healthy men at rest^8^. Additionally, a recent study has identified *DMRT3* expression in omental vs aSAT as a novel marker for the development of insulin resistance^38^. Therefore, *DMRT2* and *DMRT3* might be novel candidate genes involved in “unhealthy” central fat accumulation. Interestingly, we showed that these genes are expressed both in adipocytes and cells of the SVF, although a trend for higher expression for *DMRT3* and *HOXA5* in adipocytes than cells of the SVF was observed. Therefore, both cell types participate in the differential gene expression between the SAT depots, especially with regards to the expression of developmental genes. However, the functional role of these genes in AT development and function remains to be elucidated.

In contrast, we found higher expression of the gene *CSN1S1* in gSAT compared to aSAT at baseline and post-exercise training. Previously known as a functionally unreported adipose-specific gene, *CSN1S1* expression was recently shown to be significantly more highly expressed in SAT (abdominal) of obese compared to lean individuals^39^. Moreover, this gene is downregulated in aSAT after weight loss as shown in morbidly obese individuals^40^. Increased *CSN1S1* is involved in immune/inflammatory responses^41^ and may also play a key role in adipogenesis and lipid metabolism^39^. In the present study, the expression of *CSN1S1* was significantly higher in adipocytes than cells of the SVF in the aSAT of our independent cohort. These findings corroborate evidence showing higher expression of *CSN1S1* in mature adipocytes compared to preadipocytes and increased expression during adipose-derived stem cell differentiation^39^. Therefore, higher expression of *CSN1S1* might be reflective of higher storage capacity in gSAT compared to aSAT.

Taken together, the unique gene expression signatures of aSAT and gSAT suggest differences in developmental processes regulating AT distribution and expandability of distinct depots. Our data add to the notion that there are intrinsic morphological and functional differences between central and lower-body fat^5,6,12^. Interestingly, from the comparison of these depots at baseline and after exercise training, 18 of the DEGs were overlapping and were enriched for anatomical structure morphogenesis. We therefore postulate that the expression of developmental genes is not affected by external/environmental stimuli (such as exercise training) once they have been set during the early life stage. Rather, these differences are maintained across the depots as previously shown in cell culture studies^9,12,13,42^. Moreover, developmental genes might be actively involved in AT function, distribution and remodelling^43^. Genome-wide association studies and meta-analysis on European individuals identified several genes within the WHR-associated loci with differential transcription between aSAT and gSAT^44^. However, except for *RSPO3*, the DEGs emerging from the present study did not overlap with these previous reports^44,45^, most probably explained by the different ethnicities.

Given the lower number of DEGs between SAT depots at baseline (15 genes) compared to the number after exercise training (318 genes), we propose that the major depot-specific differences reflect the heterogeneous capacity of SAT to adapt to behavioural (or environmental) changes, such as exercise training. This hypothesis is supported by a recent study showing that fat depots (e.g. SAT, VAT, liver, perirenal) respond with a highly variable alteration in fat mass during a weight loss intervention (dietary and physical activity)^46^. The depot-specific AT response to exercise training could provide insight into metabolic and signalling pathways implicating fat distribution in the development of metabolic disorders. We therefore investigated the specific response of each SAT depot to exercise training and found expression changes in different sets of genes and associated biological processes. Notably, the expression levels of only three genes commonly changed in both depots. This suggests differential regulatory mechanisms involved in cellular and endocrine function. Specifically, the DEGs in gSAT were mostly enriched for immune and inflammatory responses (driven by upregulated genes), suggesting increased inflammatory processes after exercise training as recently shown in this cohort^47^. This is further supported by our finding of motif analyses, showing that the enriched transcription factors were associated with immune system response and pro-inflammation for higher DEGs in gSAT vs aSAT after exercise training. Exercise training can activate macrophage production of cytokines, leading to the modulation of monocyte chemotaxis, differentiation into macrophages and activation^48^. In addition, adipocytes readily respond to exercise training by modulating the transcriptomic response of pro-inflammatory genes^48^. Therefore, an increased inflammatory response is likely to originate both from adipocytes and AT immune cell signalling. In line with this, both cell types can display similar transcriptomic profiles especially when the macrophages engulf lipids^49^.

The increased inflammatory response seems to be contradictory, given the extensively reported association between AT inflammation and the development of metabolic diseases in obese humans and mouse models^49–51^ and decreased white AT inflammation after exercise training^52^. However, elevated immune response and activation of inflammatory pathways have been shown in SAT after acute exercise sessions^48,53^; and no studies have investigated the specific adaptations in gSAT in response to a long-term exercise training. The upregulation of inflammatory-related response could reflect structural changes of AT and adaptations in the early phase of the training, which is downregulated once structural changes are established^48^. However, the beneficial effect of exercise training on AT physiology is the product of continuous changes induced by repeated transient acute exercise bouts^54^. Hence the observed upregulation of immune and inflammatory responses after the present exercise training program mirrors a cumulative response of single exercise bouts. Notably, the sample analyzed in the present study were collected at least 48–72 hours after the last exercise training session to exclude the potential acute effects of the last bout of exercise training. Furthermore, these women have never completed structured exercise training before participating in this program, supporting our hypothesis that sustained inflammatory activation after 12-week exercise training is associated with AT reorganization and remodeling^47^. Consistently, the identified DEGs in gSAT in response to this combined aerobic and resistance exercise training intervention were also involved in biological processes regulating lipid metabolism and plasma lipoprotein particle levels and remodelling (e.g. *MMP9*, *SPP1*, *APOC1*, *ITGAX* and *PLA2G7*). Further, these DEGs were also enriched for transcription factors complex (AP-1) regulating cell proliferation, differentiation and transformation in response to cytokine production. Changes in metabolic homeostasis such as improved body composition or reduced adiposity can trigger immune and inflammatory processes^53^. For instance, the stimulation of lipolysis and enhanced fat mobilization in AT after exercise training was shown to be promoted by enhanced production of pro-inflammatory cytokines, such as IL-6^55^. Furthermore, pro-inflammatory signalling in adipocytes mediates angiogenesis and extracellular matrix (ECM) remodelling^56,57^ and the overall reduction of stored triglycerides in response to exercise training have been shown to precede an extensive remodelling of AT^58,59^. In the present study, we showed a higher expression of *MMP9* in the cells from the SVF compared to adipocytes in our independent cohort of European men and women with obesity. Therefore, higher expression of *MMP9* in response to exercise training may reflect adaptations of cells within the SVF rather than an adipocytes-specific response. This is in agreement with the hypothesis of ECM modification in gSAT after exercise training, indicative of gynoid fat remodeling^47^.

Surprisingly, in aSAT most of the DEGs were enriched for muscle-associated processes (e.g. *ACAT1, TCAP, MYL2, MYH7, MYBPC*). Similar muscle-related gene expression profiles were found for the first time in brown adipose of high fat diet-induced obesity-resistant rats (e.g. *TNNT3*, *ACTA1*, *ACTN3*, *MYLPF*), but their functional role in AT has not been clarified^60^. As whole AT was used in the present study, we cannot exclude the possibility that these genes were expressed from non-adipocytes/non-immune cells. Subsequent investigations on isolated cell fractions, or single-cell RNA sequencing approaches, as well as cell culture experiments, are required to further elucidate the mechanistic link between exercise training and signature changes of AT.

Our analyses were limited by the amount of material we could obtain from the SAT biopsies. Histological, as well as extensive proteome analyses, could not be performed and we can therefore not conclude whether gene expression differences were translated at the protein level. Moreover, the lack of a normal-weight control as a comparison group prevented us from concluding whether our findings are relevant pathways related to obesity and exercise training rather than an adaptation to the obese state. As the detection of DEGs was based on statistical methods, further validation is needed to rule out the possibility of false positives. Due to the small sample of women included in this study, the data may not be representative of African women and cannot be directly translated to other groups or individuals with different phenotypes and/or age, gender, socioeconomic and/or health status. Nevertheless, records on AT transcriptome of African populations are rare and this is one of the first studies providing unique data on SAT depot-specific gene expression profiles and adaptation to exercise training in black African women with obesity. This research work generated hypotheses about novel candidate genes potentially implicated in the relationship between body fat distribution, AT adaptation and metabolic status. Further investigations in more representative samples of black Africans, including normal-weight groups and men are needed to validate these findings.

In conclusion, the current study demonstrates differential gene expression profiles of SAT depots (gluteal and abdominal) in African women with obesity. While the differences at baseline suggest dissimilar developmental origin, the exercise training intervention unmasked a heterogeneous response of different SAT depots to an external stimulus. The specific functional and metabolic responses could be related to intrinsic differences in the expression of developmental genes set at an early-life stage in each AT depot. These were represented by an upregulation of the immune response and inflammatory processes, lipid metabolism and tissue remodelling in gSAT and surprisingly, mainly muscle-related processes in aSAT. Importantly, we identified four genes (*CSN1S1*, *DMRT2*, *DMRT3* and *HOXA5*) as novel candidate genes of body fat distribution pattern in Africans, whose biological function and implication in the pathogenesis of obesity-associated metabolic diseases remain to be unravelled.

## Methods

### Study design

This is a sub-study from a previously reported study using exercise training as a model to investigate the mechanisms underlying insulin resistance in a cohort of black South African women^61^. The general methods have been extensively reported^61^ and are only briefly described here. The study protocol was designed in accordance with the principles of the Helsinki Declaration with approval from the Human Research Ethics Committee of University of Cape Town (HREC REF: 827/2016) and registered to the Pan African Clinical Trial Registry (trial registration: PACTR201711002789113). Participants signed a written informed consent before enrollment in this study. They were included if both their parents were of *isiXhosa* (black African) ancestry, if they had a stable weight (<5% change from maximum weight for the last 6 months), no known metabolic or inflammatory diseases (e.g. HIV tuberculosis, active hepatitis, or rheumatoid arthritis). They were not taking any medications, non-smokers, not pregnant or lactating and were using injectable contraception (depot medroxyprogesterone acetate, 400 mg).

### Participants’ selection and Intervention

Twelve of the 20 participants from the parent study were included in this study. The selection was based on the greatest reduction in body weight in response to the exercise training intervention, as well as sample availability. Although weight loss was not the main outcome of that study, the participants with the greatest weight loss were specifically selected to maximize the responses observed in the SAT depots to exercise training. The women performed 12 weeks of supervised exercise training consisting of combined aerobic and resistance training (60–70% heart rate; HR_peak_) progressing from 40 to 60 min, 4 days per week by a trained facilitator. The exercise training consisted of cardiovascular exercises in the form of aerobic dance, running, skipping, and stepping that was performed at a moderate-vigorous intensity of 75–80% HR_peak_. Participants used their body weight for resistance training and progressed to the use of equipment subsequently (e.g. resistance bands and light free weights). Attendance was recorded at each training session and participants wore a heart rate monitor (Polar A300, Kempele, Finland) to monitor the exercise intensity. Participants were instructed to maintain their usual dietary intake throughout the 12-week intervention period, which was assessed monthly to ensure the maintenance of usual dietary intake as previously described^62^. Before and following the 12-week intervention, body composition, cardio-respiratory fitness were determined and adipose tissue samples were collected from abdominal and gluteal SAT for the subsequent analysis of gene expression as detailed below.

To dissect the contribution of cell type to whole adipose tissue gene expression we included an independent cohort of 21 women and 8 men of European Ancestry, with a mean age of 42.2 ± 12.9 years and mean BMI 49.9 ± 8.4 kg/m^2^. From this cohort, adipocytes and cells from the stromal vascular fraction (SVF) were isolated from paired abdominal SAT samples as described previously^63^. Abdominal SAT was collected during open or laparoscopic abdominal surgery^64^ and immediately frozen in liquid nitrogen and stored at −80 °C. The study was approved by the Ethics Committee of the University of Leipzig (approval no: 159‐12‐21052012) and performed in accordance with the Declaration of Helsinki. All participants gave written informed consent before taking part in this study.

### Body composition

Basic anthropometry (weight, height, waist and hip circumference) was measured. Whole-body composition (including fat mass and fat-free soft tissue mass) and regional body fat distribution (gynoid and android fat mass) were determined by dual-energy X-ray absorptiometry (DXA; Discovery-W, software version 12.7.3.7; Hologic, Bedford, MA). VAT and SAT volumes were measured by magnetic resonance imaging (MRI; 3-Tesla Skyra Whole-body Human), as previously described^65^.

### Cardiorespiratory fitness

Cardiorespiratory fitness was determined by measuring peak oxygen consumption (VO_2peak_) using a walking treadmill-based (C, Quasar LE500CE, HP Cosmos, Nussdorf-Traunstein, Germany) graded exercise test that increased in gradient by 2% every minute until 16%, followed by an alternate increase in speed (0.5 km/hour) and gradient (1%) until volitional exhaustion, as previously reported^61^. Pulmonary gas exchange was measured by determining O_2_ and CO_2_ concentrations and ventilation to calculate VO_2_ consumption and respiratory exchange ratio (RER) using a metabolic gas analysis system (CPET, Cosmed, Rome Italy)^61^.

### Adipose tissue biopsies

Before and after exercise training, whole-tissue samples from the aSAT and gSAT depots were collected by mini-liposuction after 4–6 h of fasting, and at least 48–72 h after the last exercise training session. These samples were subsequently used for RNA extraction and evaluation of the gene expression using a microarray technique.

### RNA extraction, reverse transcription and mRNA expression

Total RNA was extracted from whole-tissue SAT, adipocytes and SVF using RNeasy Lipid tissue Mini kit according to the manufacturer’s protocol (Qiagen Ltd, Germantown, MD, USA). To improve the quality and yield of the microarray probes, RNA was re-purified using RNeasy mini columns (Qiagen, Hilden, Germany). The integrity and concentration of the purified RNA was verified on an Agilent Fragment Analyzer (Agilent Technologies, Palo Alto, CA, USA) using the RNA Kit (Agilent Technologies) according to the manufacturer’s instructions.

For the mRNA expression analysis of selected DEGs, 1 µg of total RNA from isolated adipocytes and SVF was reverse-transcribed using High-Capacity cDNA Reverse Transcription Kit with RNase inhibitors (Thermofisher, Darmstadt, Germany). TaqMan probe‐based quantitative real‐time polymerase chain reaction (RT-PCR) was performed on cDNA samples using the QuantStudio 6 Flex Real‐Time PCR System (Thermofisher, Darmstadt, Germany). A standard curve was constructed for each primer-probe set using a serial dilution of cDNA pooled from all samples. The expression of *HOXA5* (Hs00430330_m1)*, DMRT2* (Hs00246364_m1), *DMRT3* (Hs00253642_m1), *RSPO3* (Hs00262176_m1), *CS1N1* (Hs00157136_m1) and *MMP9* (Hs00957562_m1) were normalized to ribosomal protein lateral stalk subunit PO (*RPLPo*; Hs99999902_m1) as endogenous gene and presented as the ratio of abundance of the gene of interest: abundance of RPLPo.

### Array data extraction and analysis

Purified RNA samples from aSAT (Pre: n = 12; Post: n = 12) and gSAT (Pre: n = 12; Post: n = 12) were analyzed before and after exercise training using Illumina Expression BeadChip (Epicentre Biotechnologies, Madison, WI, USA). One array per tissue sample, for each SAT depot and for each time point (i.e. 4 arrays per participant × 12 participants) was used for the gene expression analysis (total of 24 arrays for gSAT samples and 24 arrays for aSAT samples). Each total RNA aliquot of 250 ng was ethanol precipitated with GlycoBlue (Invitrogen) as a carrier and dissolved at a concentration of 100–150 ng/µl before probe synthesis using the TargetAmp-Nano Labeling Kit for Illumina Expression BeadChip. 750 ng of cRNA were hybridized to Human HT-12 v4 Expression BeadChips (Illumina, San Diego, CA, USA) and scanned on the Illumina iScan instrument according to the manufacturer’s specifications. Raw expression intensities of 47,323 probes were extracted using Illumina GenomeStudio (Version 1.9.0) and the normalization, background correction and analysis were executed using R statistic software (version 3.6.1).

### Statistical analysis

#### Participants’ characteristics

For the participants’ characteristics, data were presented as mean ± standard deviation. After the test of normality (Shapiro-Wilks test) and equality of variances (Barttlets’ test), the changes in cardiorespiratory fitness, body composition and dietary intake in response to exercise training were analyzed using a paired *t-test*. Significance levels were set at p < 0.05 and the analyses were performed using Stata Software version 13.1 (StataCorp, College Station, Texas 77845 USA).

#### mRNA expression analysis

The data were expressed as mean ± standard error of the mean. *Wilcoxon’s* test was performed to determine differences in the mRNA expression levels between adipocytes and SVF using Prism Version 6.05 (GraphPad Software, Inc., La Jolla, CA). The significant level was set to a p-value < 0.05.

#### Data quality control and differential gene expression analysis

The gene expression profile of aSAT and gSAT samples were processed from the raw data obtained from Illumina GenomeStudio following the protocol of Ritchie *at al*.^66^. After background correction, normalization, and log_2_ transformation (with neqc function), the probes annotated as “No match” or “Bad” were removed from the analysis. However, before removing, the 53 genes annotated as ‘Bad” but with high average expression intensities (>12) were further investigated. Forty-five were repetitive Alu-elements, and the remaining were ribosomal proteins, EEF1A1 (eukaryotic translation elongation factor), and FTH1 (Ferritin heavy chain) all of which were encoded at multiple genomic loci, making further analysis questionable.

The filtered expression data were subsequently used for differential gene expression analysis across the two depots, separately for each time point. We fitted a linear model and computed the moderated t-statistics, moderated F-statistics, and log-odds for the pairwise contrasts by empirical Bayes from the limma package^67^. This comprised correction for multiple testing (false discovery rate; FDR). Differential expression was investigated between aSAT and gSAT at baseline and post-training, as well as within each fat depot before and after exercise training. Genes were identified as differentially expressed when the difference in the expression intensities were equal or higher than 33% (i.e. fold change (FC) ≤ 0.67 for lower/downregulated genes and FC ≥ 1.5 for higher/upregulated genes) or an absolute log2 fold change (|LFC | ) ≥ 0.58, with a p-value ≤ 0.05.

#### Gene Set Enrichment and In Silico Promotor Analyses

Enriched biological processes and functional pathways represented by the identified DEGs were obtained from STRING^68^. Subsequently, the number of expected genes in a random set of the same size were compared to the number of observed DEGs assigned to a specific enriched GO-term. The DEGs were subsequently analyzed for over-represented motifs in their promoter regions using HOMER (hypergeometric optimization of motif enrichment) v4.10^69^. Detailed method and data processing of each comparison set (aSAT vs gSAT in both time point, aSAT pre- vs post-exercise training and gSAT pre- vs post-exercise training are provided in the supplementary document.

## Supplementary information

Supplementary Information.

Supplementary Information2.

Supplementary Information3.
