# Supplementary material for: Distinct abdominal and gluteal adipose tissue transcriptome signatures are altered by exercise training in African women with obesity
Source: Sci Rep. 2020 Jun 24;10:10240. doi: 10.1038/s41598-020-66868-z (PMC7314771; doi:10.1038/s41598-020-66868-z)
Supplement: Supplementary file 1 — Supplementary Information. [file 41598_2020_66868_MOESM1_ESM.docx]

*MANUSCRIPT TITLE:*

**Distinct abdominal and gluteal adipose tissue transcriptome signatures are altered by exercise training in African women with obesity**

***AUTHORS:***

Pamela A. Nono Nankam^1,2*^, Matthias Blüher^2,3^, Stephanie Kehr^4^, Nora Klöting^2,3^, Knut Krohn^5^, Kevin Adams^1^, Peter F. Stadler^4^, Amy E. Mendham^1,6^, Julia H. Goedecke^1,6^

***AFFILIATIONS:***

^1^Division of Exercise Science and Sports Medicine, Department of Human Biology, University of Cape Town, Cape Town, South Africa

^2^Department of Endocrinology, Faculty of Medicine, University of Leipzig, Leipzig, Germany

^3^Helmholtz Institute for Metabolic, Obesity and Vascular Research (HI-MAG) of the Helmholtz Zentrum München at the University of Leipzig and University Hospital Leipzig

^4^Bioinformatics Group, Department of Computer Science, and Interdisciplinary Center for Bioinformatics, University of Leipzig, Germany

^5^Core Unit DNA-Technologies, Medical Faculty, University Leipzig, Leipzig, Germany

^6^Non-communicable Diseases Research Unit, South African Medical Research Council

Tygerberg, Cape Town, South Africa.

***Correspondence to:** pamela.nononankam@medizin.uni-leipzig.de

# ***In Silico Promotor Analysis***

# **Methods**

Additionally, the DEGs were analyzed for over-represented motifs in their promoter regions using HOMER (Hypergeometric Optimization of Motif EnRichment) v4.10 [1]. The analysis was done for all DEG contrast, and separately for up- and down-regulated gene sets respectively, if the number of genes in both sets exceeds 50 genes. For the HOMER computation, the default parameters were kept, thus considering the region of -300bp/50bp as putative promoter regions. As the background gene set of all genes on the array were considered, only the motifs present in at least 5% of the target genes were considered. A p-value cut-off of 1e^-2^ (known motifs) and 1e^-9^ (de novo motifs) were chosen for over-represented motifs. The alignment of the 'best match motifs' were manually inspected to reject false positive matches. The best matches were only reported when the consensus sequence matched the core region of the suggested binding motif. Detailed data processing of each comparison set (aSAT vs gSAT in both time point, aSAT pre- vs post-exercise training and gSAT pre- vs post-exercise training) as well as the outcome from the HOMER analysis are provided below.

# **Abdominal SAT vs gSAT at baseline**

The set of DEGs between both fat depots included only 15 genes, making motif enrichment analysis unreliable. Each of the four identified known motifs is only present in four promoter sequences. Given the shortness of the motifs and the high degree of freedom in the according position weight matrices (PWM) no results are reported for putative transcription factor binding sites (TFBS) at baseline.

# **Abdominal SAT vs gSAT after exercise training**

Enrichment for TFBS in promoters’ regions was done for higher and lower DEGs (in aSAT vs gSAT), separately.

*Known results*: In the lower expressed genes in aSAT vs gSAT, an enrichment of RUNX binding site, an E-box promoter element and USF1 binding site was found. RUNX (Runt‐domain family of transcription factors) is essential for haemopoiesis. In humans, granulocyte macrophage colony-stimulating factor (GM-CSF or CSF2; pro-inflammatory cytokine) gene is a key target of regulation by RUNX1. GM-CSF (produced by activated T cells and mast cells) is induced by stimuli that activate the immune system [2]. RUNX sites (RUNX1) have also been involved in the transcriptional regulation and activation of the macrophage inflammatory protein‐1α (MIP-1α) promoter 1 [3].

The E-box motif CCGGTCACGTGA and the TFBS for upstream stimulatory factor 1 (USF1) are almost identical. The USF1 motif is shorter and starts after the leading CC. Therefore, E-box can also act as an USF1 binding site [4]. The transcription factor USF1 is linked to familial combined hyperlipidemia (FCHL) and associated to the metabolism of BAT in mice [5].

In higher expressed genes in aSAT vs gSAT, nuclear factor 1 (NF1)-half-site and lymphoma related factor (LRF, aka ZBTB7A) binding motifs are predicted to be enriched. LRF represses a variety of genes and has previously been associated to adipogenesis [6] and glycolysis [7]. However, in the union of differential expressed genes the binding site for transcription factor STAT6 is found enriched. STAT6 is associated with the IL4 mediated immune system responses to cytokines and growth factors.

*De novo results:* Among the enriched *de novo* motifs in the genes with lower expression in aSAT vs gSAT, two are similar to binding motifs of known transcription factors (TF): ZNF467 has been found involved in adipocyte differentiation in mice and humans [8, 9], and PRDM1 is associated to immune system responses [10, 11]. Additionally, two enriched motifs that have no obvious similarity to known TFBS are detected in the union of higher and lower DEGs. No enriched *de novo* motif where found in for genes with higher expression in aSAT vs gSAT.

***Supplementary Table S5***: HOMER known motif enrichment results of genes with lower expression in aSAT vs. gSAT after training.

| **Motif** | **Name** | **P-val** | **q-val** | **# Target Seq** | **% of Targets Seq** | **# Bkg Seq** | **% of Bkg Seq** |
| --- | --- | --- | --- | --- | --- | --- | --- |
| 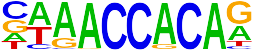 | **RUNX** (Runt) | 1e-2 | 1.0000 | 19.0 | 15.83% | 1186.9 | 7.89% |
| 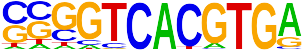 | **E-box** (bHLH) | 1e-2 | 1.0000 | 11.0 | 9.17% | 525.5 | 3.49% |
| 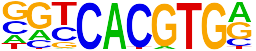 | **USF1** (bHLH) | 1e-2 | 1.0000 | 20.0 | 16.67% | 1414.1 | 9.40% |

***Supplementary Table S6:*** HOMER known motif enrichment results of genes with higher expression in aSAT vs gSAT after training.

| **Motif** | **Name** | **P-val** | **q-val** | **# Target Seq** | **% of Targets Seq** | **# Bkg Seq** | **% of Bkg Seq** |
| --- | --- | --- | --- | --- | --- | --- | --- |
| 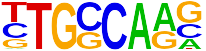 | **NF1-halfsite** (CTF) | 1e-2 | 1.0000 | 44.0 | 35.48% | 3794.5 | 24.68% |
| 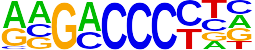 | **LRF** (Zf) | 1e-2 | 1.0000 | 60.0 | 48.39% | 5759.2 | 37.46% |

***Supplementary Table S7:*** HOMER known motif enrichment results of all DEG between aSAT and gSAT after training.

| **Motif** | **Name** | **P-val** | **q-val** | **# Target Seq** | **% of Targets Seq** | **# Bkg Seq** | **% of Bkg Seq** |
| --- | --- | --- | --- | --- | --- | --- | --- |
| 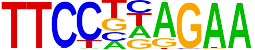 | **STAT6**(Stat) | 1e-2 | 1.0000 | 25.0 | 10.25.88% | 871.4 | 5.64% |

***Supplementary Table S8:*** HOMER *de novo* motif enrichment results of genes with lower expression in aSAT vs. gSAT after training.

| **Motif** | **P-val** | **% of Targets** | **% of Bkg** | **STD(Bg STD)** | **Best Match** |
| --- | --- | --- | --- | --- | --- |
| 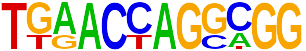 | 1e-12 | 6.67% | 0.06% | 88.1bp (101.6bp) | undefined |
| 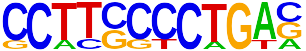 | 1e-9 | 9.17% | 0.61% | 106.4bp (115.9bp) | **ZNF467** (Zf) |
| 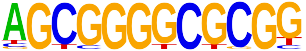 | 1e-9 | 10.00% | 0.81% | 82.2bp (88.7bp) | undefined |
| 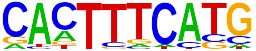 | 1e-9 | 16.67% | 2.97% | 88.8bp (106.9bp) | **PRDM1** |

***Supplementary Table S9:*** HOMER *de novo* motif enrichment results of all DEG between aSAT and gSAT after training.

| **Motif** | **P-val** | **% of Targets** | **% of Bkg** | **STD(Bg STD)** | **Best Match** |
| --- | --- | --- | --- | --- | --- |
| 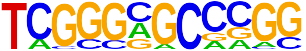 | 1e-11 | 13.11% | 2.94% | 94.3bp (103.9bp) | undefined |
| 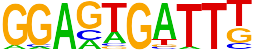 | 1e-10 | 11.48% | 2.37% | 106.3bp (98.7bp) | undefined |

# **Gluteal SAT pre- vs post-exercise training**

The number of downregulated genes in gSAT after exercise training was too low to perform separate enrichment analysis of TFBS for up and down regulated genes. Therefore, we combined both sets for one analysis. The enriched motifs found with HOMER belong to or interact with transcription factor complex AP-1. Most of the predicted known and *de novo* motifs resemble binding motifs of Fos-related factors (leucine-zipper-proteins) or the JUN family. By dimerization, both form the AP-1 complex. Additionally, one predicted over-represented binding motif is for activation transcription factor 3 (Atf3), which belongs to the cAMP responsive element binding (CREB) class interacts with JUN related factors, and BATF mediates the dimerization of FOS and JUN factors to form the AP-1 transcription factor (TF) complex. Worth noting, all predicted motifs are very similar and share a consensus TGASTCA [12].

The AP-1 TF complex is a regulator of cell proliferation, differentiation and transformation in response to stimuli like cytokine, stress, growth factor, or infection [13]. Additionally, predicted enriched known motifs are binding sites for MITF and VDR.

***Supplementary Table S10***: HOMER known motif enrichment results of DEG in gSAT before and after training.

| **Motif** | **Name** | **P-val** | **q-val** | **# Target Seq** | **% of Targets Seq** | **# Bkg Seq** | **% of Bkg Seq** |
| --- | --- | --- | --- | --- | --- | --- | --- |
| 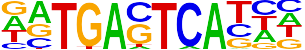 | **Atf3** (bZIP) | 1e-3 | 0.3459 | 10.0 | 20.41% | 825.7 | 6.25% |
| 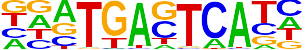 | **Fra1** (bZIP) | 1e-2 | 0.3459 | 9.0 | 18.37% | 727.7 | 5.51% |
| 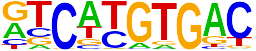 | **MITF** (bHLH) | 1e-2 | 0.5623 | 14.0 | 28.57% | 1761.8 | 13.34% |
| 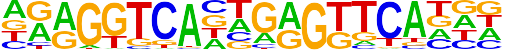 | **VDR** (NR) | 1e-2 | 0.5623 | 6.0 | 12.24% | 410.6 | 3.11% |
| 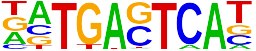 | **BATF** (bZIP) | 1e-2 | 0.7614 | 8.0 | 16.33% | 799.1 | 6.05% |

***Supplementary Table S11***: HOMER *de novo* motif enrichment results of DEG in gSAT before and after training.

| Motif | P-val | % of Targets | % of Bkg | STD(Bg STD) | Best Match |
| --- | --- | --- | --- | --- | --- |
| 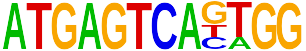 | 1e-9 | 8.16% | 0.00% | 95.0bp (0.0bp) | **JUND** |

# **Abdominal SAT pre- vs post-exercise training**

Similar to the analysis in the gSAT, we considered the union of up- and down-regulated genes in aSAT in response to exercise training to identify enriched motifs, as the number of downregulated genes was only 22. Four known TFBS are found in the promoter regions of the DEGs. The enriched CarG-box DNA motif is bound by the SRF-MRTFA nuclear complex via the serum response factor (SRF). HOMER also identifies the binding site of Mef2b to be enriched. This TF is a paralog of SRF and palys diverse roles in cell growth, survival and apoptosis via the p38 MAPK signalling.

One of the predicted *de novo* motifs is similar to the consensus sequence of the Smad2 binding site. Smad2 is a regulator of multiple cellular processes, such as cell proliferation, apoptosis and differentiation.

***Supplementary Table S12***: HOMER known motif enrichment results of DEGs in aSAT before and after training.

| **Motif** | **Name** | **P-val** | **q-val** | **# Target Seq** | **% of Targets Seq** | **# Bkg Seq** | **% of BkgSeq** |
| --- | --- | --- | --- | --- | --- | --- | --- |
| 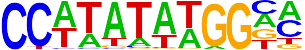 | **CarG** (MADS) | 1e-3 | 0.1605 | 10.0 | 15.87% | 624.4 | 4.33% |
| 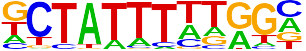 | **Mef2b** (MADS) | 1e-2 | 0.5623 | 13.0 | 20.63% | 1248.9 | 8.65% |
| 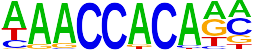 | **RUNX1** (Runt) | 1e-2 | 0.9324 | 15.0 | 23.81% | 1758.0 | 12.18% |
| 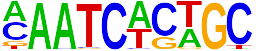 | **Gfi1b** (Zf) | 1e-2 | 0.9324 | 11.0 | 17.46% | 1137.7 | 7.88% |

***Supplementary Table S13***: HOMER *de novo* motif results of DEGs in aSAT before and after training.

| **Motif** | **P-val** | **% of Targets** | **% of Bkg** | **STD(Bg STD)** | **Best Match** |
| --- | --- | --- | --- | --- | --- |
| 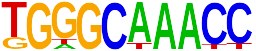 | 1e-10 | 14.29% | 0.43% | 96.5bp (125.5bp) | undefined |
| 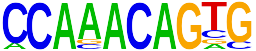 | 1e-9 | 14.29% | 0.63% | 76.3bp (115.1bp) | **Smad2** (MAD) |

**References**

1. Heinz, S., et al., *Simple combinations of lineage-determining transcription factors prime cis-regulatory elements required for macrophage and B cell identities.* Mol Cell, 2010. **38**(4): p. 576-89.

2. Bowers, S.R., et al., *Runx1 binds as a dimeric complex to overlapping Runx1 sites within a palindromic element in the human GM-CSF enhancer.* Nucleic Acids Res, 2010. **38**(18): p. 6124-34.

3. Bristow, C.A. and P. Shore, *Transcriptional regulation of the human MIP-1alpha promoter by RUNX1 and MOZ.* Nucleic Acids Res, 2003. **31**(11): p. 2735-44.

4. Ito, Y., et al., *NF-Y and USF1 transcription factor binding to CCAAT-box and E-box elements activates the CP27 promoter.* Gene, 2011. **473**(2): p. 92-9.

5. Laurila PP, S.J., Kooijman S, Forsström S, Boon MR, Surakka I, et al., *USF1 deficiency activates brown adipose tissue and improves cardiometabolic health.* Science Translational Medicine, 2016. **8**(323): p. 323ra13.

6. Laudes, M., et al., *Role of the POZ zinc finger transcription factor FBI-1 in human and murine adipogenesis.* J Biol Chem, 2004. **279**(12): p. 11711-8.

7. Liu, X.S., et al., *Somatic human ZBTB7A zinc finger mutations promote cancer progression.* Oncogene, 2016. **35**(23): p. 3071-8.

8. Quach, J.M., et al., *Zinc finger protein 467 is a novel regulator of osteoblast and adipocyte commitment.* J Biol Chem, 2011. **286**(6): p. 4186-98.

9. Gluscevic, M., et al., *Functional expression of ZNF467 and PCBP2 supports adipogenic lineage commitment in adipose-derived mesenchymal stem cells.* Gene, 2020. **737**: p. 144437.

10. Smith, M.A., et al., *PRDM1/Blimp-1 controls effector cytokine production in human NK cells.* J Immunol, 2010. **185**(10): p. 6058-67.

11. Fu, S.H., et al., *New insights into Blimp-1 in T lymphocytes: a divergent regulator of cell destiny and effector function.* J Biomed Sci, 2017. **24**(1): p. 49.

12. Karin, M., Liu, Zg., Zandi, E., *AP-1 function and regulation.* Curr Opin Cell Biol., 1997. **9**(2): p. 240‐246.

13. Papoudou-Bai, A., et al., *Expression patterns of the activator protein-1 (AP-1) family members in lymphoid neoplasms.* Clin Exp Med, 2017. **17**(3): p. 291-304.
