# Supplementary material for: Distinct abdominal and gluteal adipose tissue transcriptome signatures are altered by exercise training in African women with obesity
Source: Sci Rep. 2020 Jun 24;10:10240. doi: 10.1038/s41598-020-66868-z (PMC7314771; doi:10.1038/s41598-020-66868-z)
Supplement: Supplementary file 2 — Supplementary Information2. [file 41598_2020_66868_MOESM2_ESM.docx]

*MANUSCRIPT TITLE:*

**Distinct abdominal and gluteal adipose tissue transcriptome signatures are altered by exercise training in African women with obesity**

***AUTHORS:***

Pamela A. Nono Nankam^1,2*^, Matthias Blüher^2,3^, Stephanie Kehr^4^, Nora Klöting^2,3^, Knut Krohn^5^, Kevin Adams^1^, Peter F. Stadler^4^, Amy E. Mendham^1,6^, Julia H. Goedecke^1,6^

***AFFILIATIONS:***

^1^Division of Exercise Science and Sports Medicine, Department of Human Biology, University of Cape Town, Cape Town, South Africa

^2^Department of Endocrinology, Faculty of Medicine, University of Leipzig, Leipzig, Germany

^3^Helmholtz Institute for Metabolic, Obesity and Vascular Research (HI-MAG) of the Helmholtz Zentrum München at the University of Leipzig and University Hospital Leipzig

^4^Bioinformatics Group, Department of Computer Science, and Interdisciplinary Center for Bioinformatics, University of Leipzig, Germany

^5^Core Unit DNA-Technologies, Medical Faculty, University Leipzig, Leipzig, Germany

^6^Non-communicable Diseases Research Unit, South African Medical Research Council

Tygerberg, Cape Town, South Africa.

***Correspondence to:** pamela.nononankam@medizin.uni-leipzig.de


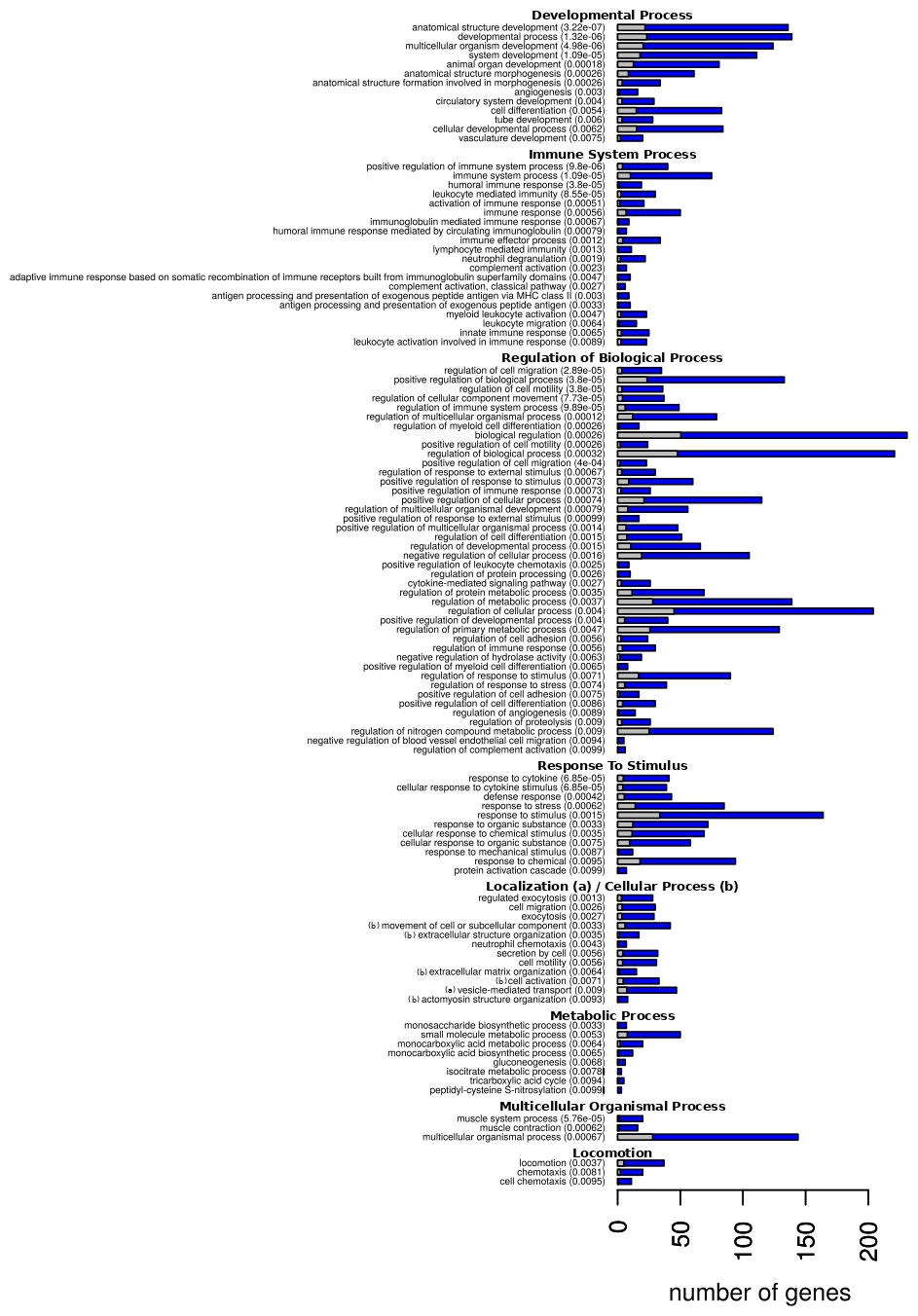
 **Supplementary figure 1:** GO term enrichment of differentially expressed genes between gluteal and abdominal SAT after (B) exercise training.

**Supplementary Figure 2**: mRNA expression in isolated adipocytes and cells of the stromal vascular fraction (SVF) normalized to *RPLPo*. Data are presented as mean ± SEM; significant differences are marked with *(P < 0.05), **(P < 0.01) or ***(P < 0.0001). AU: arbitrary units; n: number of pairs of adipocytes vs SVF.

**
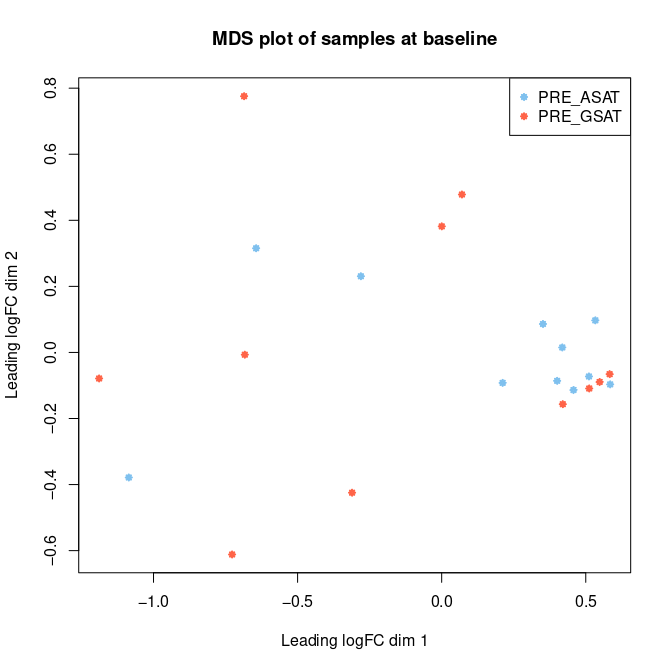
** **
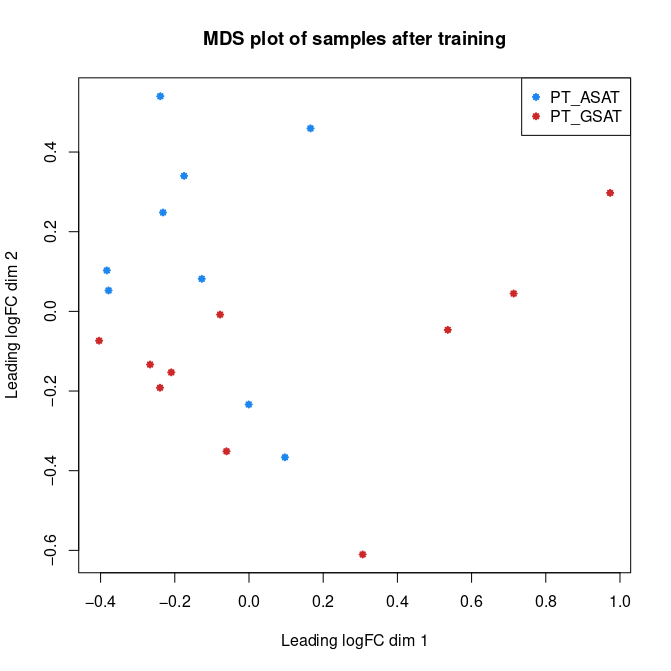
**

**
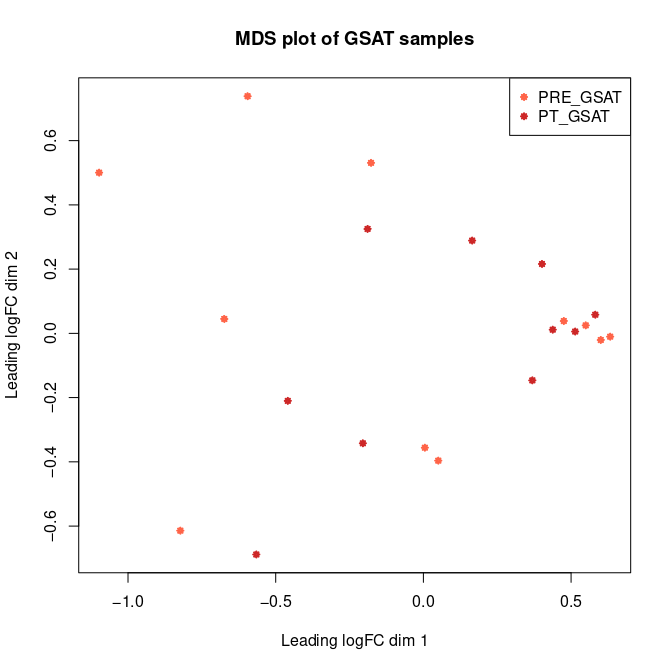
** **
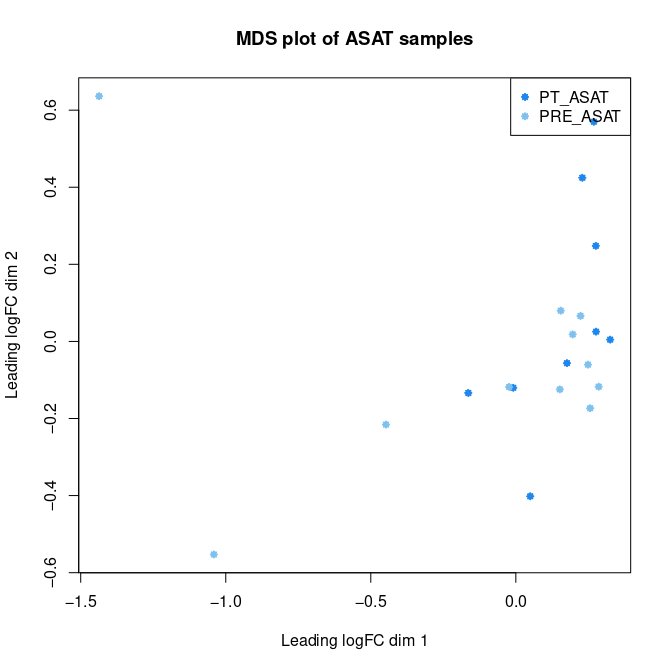
**

**Supplementary Figure 3**: Multidimensional scaling (MDS) plots of all samples, at both time points and in each adipose depot. FC: fold change; GSAT: gluteal subcutaneous adipose tissue; ASAT: abdominal subcutaneous adipose tissue; PRE: before exercise training; PT: post-exercise training.
