# Supplementary material for: Distinct abdominal and gluteal adipose tissue transcriptome signatures are altered by exercise training in African women with obesity
Source: Sci Rep. 2020 Jun 24;10:10240. doi: 10.1038/s41598-020-66868-z (PMC7314771; doi:10.1038/s41598-020-66868-z)
Supplement: Supplementary file 3 — Supplementary Information3. [file 41598_2020_66868_MOESM3_ESM.docx]

*MANUSCRIPT TITLE:*

**Distinct abdominal and gluteal adipose tissue transcriptome signatures are altered by exercise training in African women with obesity**

***AUTHORS:***

Pamela A. Nono Nankam^1,2*^, Matthias Blüher^2,3^, Stephanie Kehr^4^, Nora Klöting^2,3^, Knut Krohn^5^, Kevin Adams^1^, Peter F. Stadler^4^, Amy E. Mendham^1,6^, Julia H. Goedecke^1,6^

***AFFILIATIONS:***

^1^Division of Exercise Science and Sports Medicine, Department of Human Biology, University of Cape Town, Cape Town, South Africa

^2^Department of Endocrinology, Faculty of Medicine, University of Leipzig, Leipzig, Germany

^3^Helmholtz Institute for Metabolic, Obesity and Vascular Research (HI-MAG) of the Helmholtz Zentrum München at the University of Leipzig and University Hospital Leipzig

^4^Bioinformatics Group, Department of Computer Science, and Interdisciplinary Center for Bioinformatics, University of Leipzig, Germany

^5^Core Unit DNA-Technologies, Medical Faculty, University Leipzig, Leipzig, Germany

^6^Non-communicable Diseases Research Unit, South African Medical Research Council

Tygerberg, Cape Town, South Africa.

***Correspondence to:** pamela.nononankam@medizin.uni-leipzig.de

**Supplementary Table S1:** *Daily energy and macronutrient intake at baseline and after the 12-week exercise training intervention*

| **Variables** | **Pre (n=12)** | **Post (n=12)** | **P-Value** |
| --- | --- | --- | --- |
| **Energy (kJ)** | 11118 (9459-14243) | 10234 (7857-14022) | 0.478 |
| **Total protein (g)** | 96.1 (84.8-110.0) | 84.5 (63.5-105.0) | 0.543 |
| **Protein (%EI)** | 13.2 (12.2-14.1) | 13.0 (12.0-14.6) | 0.976 |
| **Total Fat (g)** | 107.5 (80.0-126.7) | 90.0 (66.1-107.0) | 0.226 |
| **Fat (% EI)** | 34.5 (32.1-39.1) | 32.1 (27.6-36.0) | 0.099 |
| **Total CHO (g)** | 334.2 (80.0-425.4) | 305.0 (268.7-424.0) | 0.660 |
| **CHO (%EI)** | 50.8 (49.0-52.6) | 52.0 (50.3-57.0) | 0.141 |

*Data presented as median (25^th^ and 75^th^ percentiles). kJ: kilojoules; %E: percentage of energy intake; CHO: carbohydrate*

**Supplementary Table S2:** *List of all differentially expressed genes between SAT depots after exercise training based on log2 fold change > 0.58 (up and down-regulated)*

| **SYMBOL** | **SEARCH_KEY** | **logFC** | **DEFINITION** |
| --- | --- | --- | --- |
| ***Higher in aSAT than gSAT*** | | | |
| HOXA5 | NM_019102.2 | -1.44 | Homo sapiens homeobox A5 (HOXA5), mRNA. |
| DMRT2 | NM_181872.1 | -1.41 | Homo sapiens doublesex and mab-3 related transcription factor 2 (DMRT2), transcript variant 1, mRNA. |
| DMRT3 | NM_021240.2 | -1.35 | Homo sapiens doublesex and mab-3 related transcription factor 3 (DMRT3), mRNA. |
| MYL2 | NM_000432.2 | -1.15 | Homo sapiens myosin, light chain 2, regulatory, cardiac, slow (MYL2), mRNA. |
| CDKN1B | NM_004064.2 | -1.10 | Homo sapiens cyclin-dependent kinase inhibitor 1B (p27, Kip1) (CDKN1B), mRNA. |
| MYH11 | NM_002474.1 | -1.07 | Homo sapiens myosin, heavy chain 11, smooth muscle (MYH11), transcript variant SM1A, mRNA. |
| ANGPT2 | NM_001118888.1 | -1.06 | Homo sapiens angiopoietin 2 (ANGPT2), transcript variant 3, mRNA. |
| LOC649841 | XM_938906.1 | -1.05 | PREDICTED: Homo sapiens similar to protein immuno-reactive with anti-PTH polyclonal antibodies (LOC649841), mRNA. |
| PARM1 | NM_015393.2 | -1.05 | Homo sapiens prostate androgen-regulated mucin-like protein 1 (PARM1), mRNA. |
| MYH7 | NM_000257.1 | -1.05 | Homo sapiens myosin, heavy chain 7, cardiac muscle, beta (MYH7), mRNA. |
| FLNC | NM_001458.2 | -1.02 | Homo sapiens filamin C, gamma (actin binding protein 280) (FLNC), mRNA. |
| FNDC1 | NM_032532.1 | -0.97 | Homo sapiens fibronectin type III domain containing 1 (FNDC1), mRNA. |
| HSPA1B | NM_005346.3 | -0.96 | Homo sapiens heat shock 70kDa protein 1B (HSPA1B), mRNA. |
| FAM129A | NM_022083.1 | -0.94 | Homo sapiens family with sequence similarity 129, member A (FAM129A), transcript variant 2, mRNA. |
| CLDN11 | NM_005602.4 | -0.93 | Homo sapiens claudin 11 (oligodendrocyte transmembrane protein) (CLDN11), mRNA. |
| MYADM | NM_138373.3 | -0.91 | Homo sapiens myeloid-associated differentiation marker (MYADM), transcript variant 2, mRNA. |
| PER2 | NM_022817.1 | -0.89 | Homo sapiens period homolog 2 (Drosophila) (PER2), mRNA. |
| LEPR | NM_001003679.1 | -0.88 | Homo sapiens leptin receptor (LEPR), transcript variant 2, mRNA. |
| NR5A2 | NM_003822.3 | -0.88 | Homo sapiens nuclear receptor subfamily 5, group A, member 2 (NR5A2), transcript variant 2, mRNA. |
| NRGN | NM_006176.1 | -0.88 | Homo sapiens neurogranin (protein kinase C substrate, RC3) (NRGN), mRNA. |
| RECK | NM_021111.1 | -0.87 | Homo sapiens reversion-inducing-cysteine-rich protein with kazal motifs (RECK), mRNA. |
| RSPO3 | NM_032784.3 | -0.87 | Homo sapiens R-spondin 3 homolog (Xenopus laevis) (RSPO3), mRNA. |
| MSL3 | NM_078629.2 | -0.85 | Homo sapiens male-specific lethal 3 homolog (Drosophila) (MSL3), transcript variant 1, mRNA. |
| ALDH1A1 | NM_000689.3 | -0.84 | Homo sapiens aldehyde dehydrogenase 1 family, member A1 (ALDH1A1), mRNA. |
| MUC6 | XM_932177.1 | -0.84 | Homo sapiens mucin 6, oligomeric mucus/gel-forming (MUC6), mRNA. |
| ATP6V1B1 | NM_001692.2 | -0.84 | Homo sapiens ATPase, H+ transporting, lysosomal 56/58kDa, V1 subunit B1 (ATP6V1B1), mRNA. |
| AFAP1L2 | NM_001001936.1 | -0.84 | Homo sapiens actin filament associated protein 1-like 2 (AFAP1L2), transcript variant 1, mRNA. |
| LOC100134648 | XM_001724681.1 | -0.83 | PREDICTED: Homo sapiens similar to hCG2024106, transcript variant 2 (LOC100134648), mRNA. |
| TUBB1 | NM_030773.2 | -0.83 | Homo sapiens tubulin, beta 1 (TUBB1), mRNA. |
| CXCL12 | NM_001033886.1 | -0.83 | Homo sapiens chemokine (C-X-C motif) ligand 12 (stromal cell-derived factor 1) (CXCL12), transcript variant 2, mRNA. |
| ARID4B | NM_016374.5 | -0.83 | Homo sapiens AT rich interactive domain 4B (RBP1-like) (ARID4B), transcript variant 1, mRNA. |
| MEF2C | NM_002397.2 | -0.82 | Homo sapiens myocyte enhancer factor 2C (MEF2C), mRNA. |
| SYT7 | NM_004200.2 | -0.81 | Homo sapiens synaptotagmin VII (SYT7), mRNA. |
| ATP8B2 | NM_020452.2 | -0.80 | Homo sapiens ATPase, class I, type 8B, member 2 (ATP8B2), transcript variant 1, mRNA. |
| C1QTNF9 | NM_178540.3 | -0.80 | Homo sapiens C1q and tumor necrosis factor related protein 9 (C1QTNF9), mRNA. |
| RFX7 | NM_022841.5 | -0.80 | Homo sapiens regulatory factor X, 7 (RFX7), mRNA. |
| LOC644852 | XM_934213.1 | -0.79 | PREDICTED: Homo sapiens hypothetical protein LOC644852, transcript variant 1 (LOC644852), mRNA. |
| HOPX | NM_139212.2 | -0.79 | Homo sapiens HOP homeobox (HOPX), transcript variant 3, mRNA. |
| MYOM1 | NM_003803.2 | -0.79 | Homo sapiens myomesin 1, 185kDa (MYOM1), transcript variant 1, mRNA. |
| C7 | NM_000587.2 | -0.79 | Homo sapiens complement component 7 (C7), mRNA. |
| EFNA1 | NM_004428.2 | -0.79 | Homo sapiens ephrin-A1 (EFNA1), transcript variant 1, mRNA. |
| TAOK1 | NM_020791.1 | -0.78 | Homo sapiens TAO kinase 1 (TAOK1), mRNA. |
| RERGL | NM_024730.2 | -0.78 | Homo sapiens RERG/RAS-like (RERGL), mRNA. |
| TMEM178 | NM_152390.1 | -0.77 | Homo sapiens transmembrane protein 178 (TMEM178), mRNA. |
| ZDHHC11 | NM_024786.1 | -0.77 | Homo sapiens zinc finger, DHHC-type containing 11 (ZDHHC11), mRNA. |
| SLCO3A1 | NM_013272.2 | -0.77 | Homo sapiens solute carrier organic anion transporter family, member 3A1 (SLCO3A1), mRNA. |
| RUNX1T1 | NM_175636.1 | -0.77 | Homo sapiens runt-related transcription factor 1; translocated to, 1 (cyclin D-related) (RUNX1T1), transcript variant 1, mRNA. |
| PHACTR2 | NM_014721.1 | -0.77 | Homo sapiens phosphatase and actin regulator 2 (PHACTR2), transcript variant 1, mRNA. |
| PAK2 | NM_002577.3 | -0.76 | PREDICTED: Homo sapiens p21 (CDKN1A)-activated kinase 2 (PAK2), mRNA. |
| TMEM154 | NM_152680.1 | -0.76 | Homo sapiens transmembrane protein 154 (TMEM154), mRNA. |
| HOXA3 | NM_153631.1 | -0.76 | Homo sapiens homeobox A3 (HOXA3), transcript variant 2, mRNA. |
| HOXB5 | NM_002147.2 | -0.76 | Homo sapiens homeobox B5 (HOXB5), mRNA. |
| PRMT2 | NM_206962.1 | -0.76 | Homo sapiens protein arginine methyltransferase 2 (PRMT2), transcript variant 1, mRNA. |
| LOC730525 | NM_199327.1 | -0.76 | PREDICTED: Homo sapiens hypothetical protein LOC730525 (LOC730525), mRNA. |
| MSL3L1 | NM_078630.1 | -0.75 | Homo sapiens male-specific lethal 3-like 1 (Drosophila) (MSL3L1), transcript variant 1, mRNA. |
| SEPT5 | NM_000407.3 | -0.75 | Homo sapiens septin 5 (SEPT5), mRNA. |
| LOC400986 | NM_001010914.1 | -0.75 | PREDICTED: Homo sapiens protein immuno-reactive with anti-PTH polyclonal antibodies (LOC400986), mRNA. |
| COL4A5 | NM_033381.1 | -0.75 | Homo sapiens collagen, type IV, alpha 5 (COL4A5), transcript variant 1, mRNA. |
| MAP3K1 | XM_042066.10 | -0.74 | Homo sapiens mitogen-activated protein kinase kinase kinase 1 (MAP3K1), mRNA. |
| NBPF8 | XM_001726946.1 | -0.74 | PREDICTED: Homo sapiens neuroblastoma breakpoint family, member 8 (NBPF8), mRNA. |
| C1orf24 | NM_052966.1 | -0.74 | Homo sapiens chromosome 1 open reading frame 24 (C1orf24), transcript variant 2, mRNA. |
| FEM1C | NM_020177.2 | -0.73 | Homo sapiens fem-1 homolog c (C. elegans) (FEM1C), mRNA. |
|  | Hs.436134 | -0.73 | ta96c03.x1 NCI_CGAP_Lu26 Homo sapiens cDNA clone IMAGE:2051908 3, mRNA sequence |
| CAST | NM_001750.4 | -0.73 | Homo sapiens calpastatin (CAST), transcript variant 9, mRNA. |
| LOC440928 | XM_942885.1 | -0.73 | PREDICTED: Homo sapiens hypothetical LOC440928 (LOC440928), mRNA. |
| ZNF462 | NM_021224.3 | -0.72 | Homo sapiens zinc finger protein 462 (ZNF462), mRNA. |
| PLCB1 | NM_182734.1 | -0.72 | Homo sapiens phospholipase C, beta 1 (phosphoinositide-specific) (PLCB1), transcript variant 1, mRNA. |
| LOC644162 | XM_933956.1 | -0.72 | PREDICTED: Homo sapiens similar to septin 7, transcript variant 4 (LOC644162), mRNA. |
| DKK3 | NM_013253.4 | -0.71 | Homo sapiens dickkopf homolog 3 (Xenopus laevis) (DKK3), transcript variant 2, mRNA. |
| SAPS2 | XM_942540.1 | -0.71 | PREDICTED: Homo sapiens SAPS domain family, member 2, transcript variant 2 (SAPS2), mRNA. |
| VPS13C | NM_018080.2 | -0.71 | Homo sapiens vacuolar protein sorting 13 homolog C (S. cerevisiae) (VPS13C), transcript variant 1B, mRNA. |
| BRD3 | NM_007371.2 | -0.71 | Homo sapiens bromodomain containing 3 (BRD3), mRNA. |
| SMARCA1 | NM_003069.2 | -0.71 | Homo sapiens SWI/SNF related, matrix associated, actin dependent regulator of chromatin, subfamily a, member 1 (SMARCA1), transcript variant 1, mRNA. |
| TM4SF18 | NM_138786.1 | -0.71 | Homo sapiens transmembrane 4 L six family member 18 (TM4SF18), mRNA. |
| MPZL2 | NM_005797.2 | -0.71 | Homo sapiens myelin protein zero-like 2 (MPZL2), transcript variant 1, mRNA. |
| C1orf71 | NM_152609.1 | -0.71 | Homo sapiens chromosome 1 open reading frame 71 (C1orf71), mRNA. |
| SNTB2 | NM_006750.2 | -0.71 | Homo sapiens syntrophin, beta 2 (dystrophin-associated protein A1, 59kDa, basic component 2) (SNTB2), transcript variant 1, mRNA. |
| IRX2 | NM_033267.2 | -0.70 | Homo sapiens iroquois homeobox 2 (IRX2), mRNA. |
| MYADM | NM_001020820.1 | -0.70 | Homo sapiens myeloid-associated differentiation marker (MYADM), transcript variant 4, mRNA. |
| LBH | NM_030915.1 | -0.70 | Homo sapiens limb bud and heart development homolog (mouse) (LBH), mRNA. |
| ETS1 | NM_005238.2 | -0.70 | Homo sapiens v-ets erythroblastosis virus E26 oncogene homolog 1 (avian) (ETS1), mRNA. |
| FNDC1 | NM_032532.1 | -0.69 | Homo sapiens fibronectin type III domain containing 1 (FNDC1), mRNA. |
| MTF2 | NM_007358.1 | -0.69 | Homo sapiens metal response element binding transcription factor 2 (MTF2), mRNA. |
| APCDD1L | NM_153360.1 | -0.69 | Homo sapiens adenomatosis polyposis coli down-regulated 1-like (APCDD1L), mRNA. |
| CPNE8 | NM_153634.2 | -0.69 | Homo sapiens copine VIII (CPNE8), mRNA. |
| STXBP6 | NM_014178.6 | -0.68 | Homo sapiens syntaxin binding protein 6 (amisyn) (STXBP6), mRNA. |
| ANGPT2 | NM_001147.1 | -0.68 | Homo sapiens angiopoietin 2 (ANGPT2), mRNA. |
| SMARCC2 | NM_003075.2 | -0.67 | Homo sapiens SWI/SNF related, matrix associated, actin dependent regulator of chromatin, subfamily c, member 2 (SMARCC2), transcript variant 2, mRNA. |
| C4orf31 | NM_024574.2 | -0.67 | Homo sapiens chromosome 4 open reading frame 31 (C4orf31), mRNA. |
| ADO | NM_032804.5 | -0.67 | Homo sapiens 2-aminoethanethiol (cysteamine) dioxygenase (ADO), mRNA. |
| DACH1 | NM_080759.3 | -0.67 | Homo sapiens dachshund homolog 1 (Drosophila) (DACH1), transcript variant 2, mRNA. |
| ALDH1A1 | NM_000689.3 | -0.67 | Homo sapiens aldehyde dehydrogenase 1 family, member A1 (ALDH1A1), mRNA. |
| FRMD3 | NM_174938.3 | -0.67 | Homo sapiens FERM domain containing 3 (FRMD3), mRNA. |
| NAP1L1 | NM_139207.1 | -0.66 | Homo sapiens nucleosome assembly protein 1-like 1 (NAP1L1), transcript variant 1, mRNA. |
| TMEM47 | NM_031442.2 | -0.66 | Homo sapiens transmembrane protein 47 (TMEM47), mRNA. |
| PSMC1 | XM_928629.1 | -0.66 | Homo sapiens proteasome (prosome, macropain) 26S subunit, ATPase, 1 (PSMC1), mRNA. |
| GALNTL1 | NM_020692.1 | -0.66 | Homo sapiens UDP-N-acetyl-alpha-D-galactosamine:polypeptide N-acetylgalactosaminyltransferase-like 1 (GALNTL1), mRNA. |
| CHD1 | NM_001270.2 | -0.66 | Homo sapiens chromodomain helicase DNA binding protein 1 (CHD1), mRNA. |
| EIF2C2 | NM_012154.2 | -0.66 | Homo sapiens eukaryotic translation initiation factor 2C, 2 (EIF2C2), mRNA. |
| BAT2D1 | NM_015172.2 | -0.66 | Homo sapiens BAT2 domain containing 1 (BAT2D1), mRNA. |
| SAFB2 | NM_014649.2 | -0.65 | Homo sapiens scaffold attachment factor B2 (SAFB2), mRNA. |
| CNN3 | NM_001839.2 | -0.65 | Homo sapiens calponin 3, acidic (CNN3), mRNA. |
| ARID4B | NM_016374.5 | -0.65 | Homo sapiens AT rich interactive domain 4B (RBP1-like) (ARID4B), transcript variant 1, mRNA. |
| TSHZ3 | NM_020856.1 | -0.65 | Homo sapiens teashirt zinc finger homeobox 3 (TSHZ3), mRNA. |
| PTRF | NM_012232.2 | -0.65 | Homo sapiens polymerase I and transcript release factor (PTRF), mRNA. |
| ULK1 | XM_942125.1 | -0.65 | Homo sapiens unc-51-like kinase 1 (C. elegans) (ULK1), mRNA. |
| SULF1 | NM_015170.1 | -0.65 | Homo sapiens sulfatase 1 (SULF1), mRNA. |
| TNRC6B | NM_001024843.1 | -0.65 | Homo sapiens trinucleotide repeat containing 6B (TNRC6B), transcript variant 2, mRNA. |
| HELZ | NM_014877.2 | -0.64 | Homo sapiens helicase with zinc finger (HELZ), mRNA. |
| CXCR7 | NM_020311.1 | -0.64 | Homo sapiens chemokine (C-X-C motif) receptor 7 (CXCR7), mRNA. |
| SERPINI1 | NM_005025.2 | -0.64 | Homo sapiens serpin peptidase inhibitor, clade I (neuroserpin), member 1 (SERPINI1), mRNA. |
|  | Hs.388347 | -0.64 | Homo sapiens mRNA; cDNA DKFZp686J0156 (from clone DKFZp686J0156) |
| ULK1 | NM_003565.1 | -0.64 | Homo sapiens unc-51-like kinase 1 (C. elegans) (ULK1), mRNA. |
| ZBTB20 | NM_015642.2 | -0.64 | Homo sapiens zinc finger and BTB domain containing 20 (ZBTB20), mRNA. |
| PTGDS | NM_000954.5 | -0.64 | Homo sapiens prostaglandin D2 synthase 21kDa (brain) (PTGDS), mRNA. |
| LOC651309 | XM_942586.1 | -0.64 | PREDICTED: Homo sapiens hypothetical protein LOC651309 (LOC651309), mRNA. |
| PCDH17 | NM_014459.2 | -0.64 | Homo sapiens protocadherin 17 (PCDH17), mRNA. |
|  | Hs.555252 | -0.64 | DA371742 BRTHA2 Homo sapiens cDNA clone BRTHA2001741 5, mRNA sequence |
| PLS3 | NM_005032.3 | -0.64 | Homo sapiens plastin 3 (T isoform) (PLS3), mRNA. |
| RAD21 | NM_006265.1 | -0.64 | Homo sapiens RAD21 homolog (S. pombe) (RAD21), mRNA. |
|  | Hs.379253 | -0.64 | Homo sapiens mRNA; cDNA DKFZp686J23256 (from clone DKFZp686J23256) |
| LOC441155 | XM_930970.1 | -0.64 | PREDICTED: Homo sapiens similar to Zinc finger CCCH-type domain-containing protein 11A, transcript variant 2 (LOC441155), mRNA. |
| C10orf140 | NM_207371.3 | -0.63 | Homo sapiens chromosome 10 open reading frame 140 (C10orf140), mRNA. |
| FAM179B | NM_015091.1 | -0.63 | Homo sapiens family with sequence similarity 179, member B (FAM179B), mRNA. |
| CFI | NM_000204.1 | -0.63 | Homo sapiens complement factor I (CFI), mRNA. |
| NFAT5 | NM_173215.1 | -0.63 | Homo sapiens nuclear factor of activated T-cells 5, tonicity-responsive (NFAT5), transcript variant 5, mRNA. |
| SENP7 | NM_001077203.1 | -0.63 | Homo sapiens SUMO1/sentrin specific peptidase 7 (SENP7), transcript variant 2, mRNA. |
| RIPK5 | NM_199462.1 | -0.62 | Homo sapiens receptor interacting protein kinase 5 (RIPK5), transcript variant 2, mRNA. |
| DMWD | NM_004943.1 | -0.62 | Homo sapiens dystrophia myotonica, WD repeat containing (DMWD), mRNA. |
| NFKBIA | NM_020529.1 | -0.62 | Homo sapiens nuclear factor of kappa light polypeptide gene enhancer in B-cells inhibitor, alpha (NFKBIA), mRNA. |
| PDE5A | NM_033437.2 | -0.62 | Homo sapiens phosphodiesterase 5A, cGMP-specific (PDE5A), transcript variant 1, mRNA. |
| C4orf32 | NM_152400.1 | -0.62 | Homo sapiens chromosome 4 open reading frame 32 (C4orf32), mRNA. |
| LRRC17 | NM_001031692.1 | -0.62 | Homo sapiens leucine rich repeat containing 17 (LRRC17), transcript variant 2, mRNA. |
| FAM13B | NM_001101800.1 | -0.62 | Homo sapiens family with sequence similarity 13, member B (FAM13B), transcript variant 2, mRNA. |
| CPE | NM_001873.1 | -0.62 | Homo sapiens carboxypeptidase E (CPE), mRNA. |
| C21orf7 | NM_020152.2 | -0.62 | Homo sapiens chromosome 21 open reading frame 7 (C21orf7), mRNA. |
| COL8A1 | NM_020351.2 | -0.61 | Homo sapiens collagen, type VIII, alpha 1 (COL8A1), transcript variant 2, mRNA. |
| ABTB1 | NM_172027.1 | -0.61 | Homo sapiens ankyrin repeat and BTB (POZ) domain containing 1 (ABTB1), transcript variant 1, mRNA. |
| SERPINE2 | NM_006216.2 | -0.61 | Homo sapiens serpin peptidase inhibitor, clade E (nexin, plasminogen activator inhibitor type 1), member 2 (SERPINE2), mRNA. |
| PDCD10 | NM_145860.1 | -0.61 | Homo sapiens programmed cell death 10 (PDCD10), transcript variant 2, mRNA. |
| EDN1 | NM_001955.2 | -0.61 | Homo sapiens endothelin 1 (EDN1), mRNA. |
|  | Hs.184721 | -0.61 | EST366269 MAGE resequences, MAGC Homo sapiens cDNA, mRNA sequence |
| KLHL28 | NM_017658.2 | -0.61 | Homo sapiens kelch-like 28 (Drosophila) (KLHL28), mRNA. |
| BST2 | NM_004335.2 | -0.61 | Homo sapiens bone marrow stromal cell antigen 2 (BST2), mRNA. |
| VCAN | NM_004385.2 | -0.61 | Homo sapiens versican (VCAN), mRNA. |
|  | Hs.571887 | -0.61 | Homo sapiens cDNA: FLJ21429 fis, clone COL04205 |
| RNF19A | NM_183419.1 | -0.61 | Homo sapiens ring finger protein 19A (RNF19A), transcript variant 1, mRNA. |
| LRRC1 | NM_018214.3 | -0.60 | Homo sapiens leucine rich repeat containing 1 (LRRC1), mRNA. |
| DOCK10 | NM_014689.1 | -0.60 | Homo sapiens dedicator of cytokinesis 10 (DOCK10), mRNA. |
| MBNL3 | NM_133486.1 | -0.60 | Homo sapiens muscleblind-like 3 (Drosophila) (MBNL3), transcript variant R, mRNA. |
| ISM1 | XM_939699.1 | -0.60 | Homo sapiens isthmin 1 homolog (zebrafish) (ISM1), mRNA. |
| KIAA1600 | NM_020940.2 | -0.60 | Homo sapiens KIAA1600 (KIAA1600), mRNA. |
| CDCA7 | NM_145810.1 | -0.60 | Homo sapiens cell division cycle associated 7 (CDCA7), transcript variant 1, mRNA. |
| C6 | NM_000065.1 | -0.59 | Homo sapiens complement component 6 (C6), mRNA. |
| DPYSL5 | NM_020134.2 | -0.59 | Homo sapiens dihydropyrimidinase-like 5 (DPYSL5), mRNA. |
| MLLT10 | NM_004641.2 | -0.59 | Homo sapiens myeloid/lymphoid or mixed-lineage leukemia (trithorax homolog, Drosophila); translocated to, 10 (MLLT10), transcript variant 1, mRNA. |
| HOXA9 | NM_152739.2 | -0.59 | Homo sapiens homeobox A9 (HOXA9), mRNA. |
| PGM5 | XM_936702.1 | -0.59 | Homo sapiens phosphoglucomutase 5 (PGM5), mRNA. |
| KIAA1267 | NM_015443.2 | -0.59 | Homo sapiens KIAA1267 (KIAA1267), mRNA. |
| TMEM200A | NM_052913.2 | -0.59 | Homo sapiens transmembrane protein 200A (TMEM200A), mRNA. |
| C17orf58 | NM_181656.1 | -0.59 | Homo sapiens chromosome 17 open reading frame 58 (C17orf58), transcript variant 2, mRNA. |
| PDPN | NM_001006625.1 | -0.59 | Homo sapiens podoplanin (PDPN), transcript variant 4, mRNA. |
| SORBS2 | NM_003603.4 | -0.59 | Homo sapiens sorbin and SH3 domain containing 2 (SORBS2), transcript variant 1, mRNA. |
| SORL1 | NM_003105.3 | -0.59 | Homo sapiens sortilin-related receptor, L(DLR class) A repeats-containing (SORL1), mRNA. |
| ZNF385D | NM_024697.1 | -0.59 | Homo sapiens zinc finger protein 385D (ZNF385D), mRNA. |
| CBX6 | NM_014292.3 | -0.59 | Homo sapiens chromobox homolog 6 (CBX6), mRNA. |
| ***Lower in aSAT than gSAT*** | | | |
| CSN1S1 | NM_001025104.1 | 1.37 | Homo sapiens casein alpha s1 (CSN1S1), transcript variant 1, mRNA. |
| SPP1 | NM_001040058.1 | 1.25 | Homo sapiens secreted phosphoprotein 1 (SPP1), transcript variant 1, mRNA. |
| LOC644936 | NR_004845.1 | 1.22 | Homo sapiens cytoplasmic beta-actin pseudogene (LOC644936), non-coding RNA. |
| IFI30 | NM_006332.3 | 1.17 | Homo sapiens interferon, gamma-inducible protein 30 (IFI30), mRNA. |
| LAPTM5 | NM_006762.1 | 1.17 | Homo sapiens lysosomal multispanning membrane protein 5 (LAPTM5), mRNA. |
| SPP1 | NM_000582.2 | 1.16 | Homo sapiens secreted phosphoprotein 1 (SPP1), transcript variant 2, mRNA. |
| FCGBP | NM_003890.1 | 1.15 | Homo sapiens Fc fragment of IgG binding protein (FCGBP), mRNA. |
| TM4SF19 | NM_138461.1 | 1.13 | PREDICTED: Homo sapiens transmembrane 4 L six family member 19, transcript variant 2 (TM4SF19), mRNA. |
| CSN1S1 | NM_001890.1 | 1.12 | Homo sapiens casein alpha s1 (CSN1S1), transcript variant 1, mRNA. |
| FOSB | NM_006732.1 | 1.10 | Homo sapiens FBJ murine osteosarcoma viral oncogene homolog B (FOSB), mRNA. |
| LOC55908 | NM_018687.3 | 1.10 | Homo sapiens hepatocellular carcinoma-associated gene TD26 (LOC55908), mRNA. |
| LOC645313 | XR_017585.2 | 1.09 | PREDICTED: Homo sapiens misc_RNA (LOC645313), miscRNA. |
| CD163 | NM_203416.1 | 1.06 | Homo sapiens CD163 molecule (CD163), transcript variant 2, mRNA. |
| APOC1 | NM_001645.3 | 1.02 | Homo sapiens apolipoprotein C-I (APOC1), mRNA. |
| G0S2 | NM_015714.2 | 1.01 | Homo sapiens G0/G1switch 2 (G0S2), mRNA. |
| LOC649679 | XM_945045.1 | 1.00 | PREDICTED: Homo sapiens similar to Tubulin beta-4q chain, transcript variant 2 (LOC649679), mRNA. |
| TUBB4Q | NM_020040.3 | 0.98 | Homo sapiens tubulin, beta polypeptide 4, member Q (TUBB4Q), mRNA. |
| CD163 | NM_203416.1 | 0.98 | Homo sapiens CD163 molecule (CD163), transcript variant 2, mRNA. |
| FCER1G | NM_004106.1 | 0.96 | Homo sapiens Fc fragment of IgE, high affinity I, receptor for; gamma polypeptide (FCER1G), mRNA. |
| FCGBP | XM_940656.1 | 0.95 | PREDICTED: Homo sapiens Fc fragment of IgG binding protein (FCGBP), mRNA. |
| MYOC | NM_000261.1 | 0.95 | Homo sapiens myocilin, trabecular meshwork inducible glucocorticoid response (MYOC), mRNA. |
| LAMB3 | NM_000228.2 | 0.94 | Homo sapiens laminin, beta 3 (LAMB3), transcript variant 1, mRNA. |
| CYR61 | NM_001554.3 | 0.93 | Homo sapiens cysteine-rich, angiogenic inducer, 61 (CYR61), mRNA. |
| MYOC | NM_000261.1 | 0.92 | Homo sapiens myocilin, trabecular meshwork inducible glucocorticoid response (MYOC), mRNA. |
| PLIN2 | NM_001122.2 | 0.92 | Homo sapiens perilipin 2 (PLIN2), mRNA. |
| TNMD | NM_022144.1 | 0.91 | Homo sapiens tenomodulin (TNMD), mRNA. |
| ITGAX | NM_000887.3 | 0.91 | Homo sapiens integrin, alpha X (complement component 3 receptor 4 subunit) (ITGAX), mRNA. |
| FPR3 | NM_002030.3 | 0.90 | Homo sapiens formyl peptide receptor 3 (FPR3), mRNA. |
| PEMT | NM_148173.1 | 0.87 | Homo sapiens phosphatidylethanolamine N-methyltransferase (PEMT), nuclear gene encoding mitochondrial protein, transcript variant 1, mRNA. |
| HLA-DQA1 | XM_936120.1 | 0.86 | PREDICTED: Homo sapiens major histocompatibility complex, class II, DQ alpha 1, transcript variant 10 (HLA-DQA1), mRNA. |
| CHI3L1 | NM_001276.2 | 0.86 | Homo sapiens chitinase 3-like 1 (cartilage glycoprotein-39) (CHI3L1), mRNA. |
| FBP1 | NM_000507.2 | 0.86 | Homo sapiens fructose-1,6-bisphosphatase 1 (FBP1), mRNA. |
| CAPG | NM_001747.2 | 0.86 | Homo sapiens capping protein (actin filament), gelsolin-like (CAPG), mRNA. |
| VSIG4 | NM_007268.1 | 0.84 | Homo sapiens V-set and immunoglobulin domain containing 4 (VSIG4), transcript variant 1, mRNA. |
| VEGFB | NM_003377.3 | 0.84 | Homo sapiens vascular endothelial growth factor B (VEGFB), mRNA. |
| LOC644237 | XR_039184.1 | 0.84 | PREDICTED: Homo sapiens misc_RNA (LOC644237), miscRNA. |
| PLA2G7 | NM_005084.2 | 0.82 | Homo sapiens phospholipase A2, group VII (platelet-activating factor acetylhydrolase, plasma) (PLA2G7), mRNA. |
| TUBB | NM_178014.2 | 0.82 | Homo sapiens tubulin, beta (TUBB), mRNA. |
| C1QC | NM_172369.2 | 0.81 | Homo sapiens complement component 1, q subcomponent, C chain (C1QC), mRNA. |
| ACP5 | NM_001611.2 | 0.80 | Homo sapiens acid phosphatase 5, tartrate resistant (ACP5), mRNA. |
| C1QB | NM_000491.2 | 0.80 | Homo sapiens complement component 1, q subcomponent, B chain (C1QB), mRNA. |
| ANKRD33 | NM_182608.2 | 0.80 | Homo sapiens ankyrin repeat domain 33 (ANKRD33), mRNA. |
| TREM2 | NM_018965.1 | 0.77 | Homo sapiens triggering receptor expressed on myeloid cells 2 (TREM2), mRNA. |
| IDH2 | NM_002168.2 | 0.77 | Homo sapiens isocitrate dehydrogenase 2 (NADP+), mitochondrial (IDH2), nuclear gene encoding mitochondrial protein, mRNA. |
| CISH | NM_145071.1 | 0.77 | Homo sapiens cytokine inducible SH2-containing protein (CISH), mRNA. |
| RBP4 | NM_006744.3 | 0.75 | Homo sapiens retinol binding protein 4, plasma (RBP4), mRNA. |
| CPVL | NM_031311.3 | 0.75 | Homo sapiens carboxypeptidase, vitellogenic-like (CPVL), transcript variant 1, mRNA. |
| SRPR | NM_003139.2 | 0.74 | Homo sapiens signal recognition particle receptor (docking protein) (SRPR), mRNA. |
| TM7SF4 | NM_030788.2 | 0.74 | Homo sapiens transmembrane 7 superfamily member 4 (TM7SF4), mRNA. |
| MRAS | NM_012219.2 | 0.73 | Homo sapiens muscle RAS oncogene homolog (MRAS), transcript variant 1, mRNA. |
| CYP4B1 | NM_000779.2 | 0.73 | Homo sapiens cytochrome P450, family 4, subfamily B, polypeptide 1 (CYP4B1), transcript variant 2, mRNA. |
| LOC100133678 | XM_001719804.1 | 0.72 | PREDICTED: Homo sapiens similar to hCG2042724 (LOC100133678), partial mRNA. |
| TUBB2C | NM_006088.5 | 0.72 | Homo sapiens tubulin, beta 2C (TUBB2C), mRNA. |
| CARS | NM_001751.4 | 0.71 | Homo sapiens cysteinyl-tRNA synthetase (CARS), transcript variant 4, mRNA. |
| ALCAM | NM_001627.2 | 0.71 | Homo sapiens activated leukocyte cell adhesion molecule (ALCAM), mRNA. |
| FTHL3 | NR_002201.1 | 0.71 | Homo sapiens ferritin, heavy polypeptide-like 3 (FTHL3), non-coding RNA. |
| DPP7 | NM_013379.2 | 0.71 | Homo sapiens dipeptidyl-peptidase 7 (DPP7), mRNA. |
| KIAA1598 | NM_018330.3 | 0.70 | Homo sapiens KIAA1598 (KIAA1598), mRNA. |
| ABCC6 | XM_936351.1 | 0.70 | Homo sapiens ATP-binding cassette, sub-family C (CFTR/MRP), member 6 (ABCC6), transcript variant 1, mRNA. |
| CXCL9 | NM_002416.1 | 0.70 | Homo sapiens chemokine (C-X-C motif) ligand 9 (CXCL9), mRNA. |
| SLC6A8 | NM_005629.1 | 0.70 | Homo sapiens solute carrier family 6 (neurotransmitter transporter, creatine), member 8 (SLC6A8), mRNA. |
| AHNAK | NM_024060.2 | 0.69 | Homo sapiens AHNAK nucleoprotein (AHNAK), transcript variant 2, mRNA. |
| DECR1 | NM_001359.1 | 0.69 | Homo sapiens 2,4-dienoyl CoA reductase 1, mitochondrial (DECR1), nuclear gene encoding mitochondrial protein, mRNA. |
| MS4A6E | NM_139249.2 | 0.69 | Homo sapiens membrane-spanning 4-domains, subfamily A, member 6E (MS4A6E), mRNA. |
| RASSF4 | NM_178145.1 | 0.68 | Homo sapiens Ras association (RalGDS/AF-6) domain family member 4 (RASSF4), mRNA. |
| CCL22 | NM_002990.3 | 0.68 | Homo sapiens chemokine (C-C motif) ligand 22 (CCL22), mRNA. |
| MAL2 | NM_052886.1 | 0.68 | Homo sapiens mal, T-cell differentiation protein 2 (MAL2), mRNA. |
| NNMT | NM_006169.2 | 0.68 | Homo sapiens nicotinamide N-methyltransferase (NNMT), mRNA. |
| FASN | NM_004104.4 | 0.68 | Homo sapiens fatty acid synthase (FASN), mRNA. |
| ZNF385A | NM_015481.1 | 0.68 | Homo sapiens zinc finger protein 385A (ZNF385A), transcript variant 3, mRNA. |
| GAPDH |  | 0.68 | Homo sapiens glyceraldehyde-3-phosphate dehydrogenase (GAPDH), mRNA. |
| IRF8 | NM_002163.2 | 0.67 | Homo sapiens interferon regulatory factor 8 (IRF8), mRNA. |
| C1QA | NM_015991.1 | 0.67 | Homo sapiens complement component 1, q subcomponent, alpha polypeptide (C1QA), mRNA. |
| LGALS12 | NM_033101.2 | 0.67 | Homo sapiens lectin, galactoside-binding, soluble, 12 (LGALS12), mRNA. |
| NCKAP1L | NM_005337.2 | 0.67 | Homo sapiens NCK-associated protein 1-like (NCKAP1L), mRNA. |
| INDO | NM_002164.3 | 0.67 | Homo sapiens indoleamine-pyrrole 2,3 dioxygenase (INDO), mRNA. |
| ATP6V0C | NM_001694.2 | 0.67 | PREDICTED: Homo sapiens ATPase, H+ transporting, lysosomal 16kDa, V0 subunit c (ATP6V0C), mRNA. |
| REEP6 | NM_138393.1 | 0.67 | Homo sapiens receptor accessory protein 6 (REEP6), mRNA. |
| FOLR2 | NM_000803.2 | 0.67 | Homo sapiens folate receptor 2 (fetal) (FOLR2), mRNA. |
| CRYBB2 | NM_000496.1 | 0.67 | Homo sapiens crystallin, beta B2 (CRYBB2), mRNA. |
| CD74 | NM_004355.2 | 0.67 | Homo sapiens CD74 molecule, major histocompatibility complex, class II invariant chain (CD74), transcript variant 2, mRNA. |
| CLMN | NM_024734.2 | 0.66 | Homo sapiens calmin (calponin-like, transmembrane) (CLMN), mRNA. |
| SAA1 | NM_199161.1 | 0.66 | Homo sapiens serum amyloid A1 (SAA1), transcript variant 2, mRNA. |
| ME3 | NM_006680.2 | 0.66 | Homo sapiens malic enzyme 3, NADP(+)-dependent, mitochondrial (ME3), nuclear gene encoding mitochondrial protein, transcript variant 2, mRNA. |
| PTPLA | NM_014241.2 | 0.66 | Homo sapiens protein tyrosine phosphatase-like (proline instead of catalytic arginine), member A (PTPLA), mRNA. |
| CTSA | NM_000308.1 | 0.66 | Homo sapiens cathepsin A (CTSA), transcript variant 1, mRNA. |
| LY86 | NM_004271.3 | 0.66 | Homo sapiens lymphocyte antigen 86 (LY86), mRNA. |
| AP1B1 | NM_145730.1 | 0.66 | Homo sapiens adaptor-related protein complex 1, beta 1 subunit (AP1B1), transcript variant 2, mRNA. |
| GPC1 | NM_002081.1 | 0.66 | Homo sapiens glypican 1 (GPC1), mRNA. |
| DMPK | NM_004409.2 | 0.66 | Homo sapiens dystrophia myotonica-protein kinase (DMPK), transcript variant 4, mRNA. |
| DAP | NM_004394.1 | 0.65 | Homo sapiens death-associated protein (DAP), mRNA. |
| GAPDH | NM_002046.2 | 0.65 | Homo sapiens glyceraldehyde-3-phosphate dehydrogenase (GAPDH), mRNA. |
| ITPK1 | NM_014216.3 | 0.65 | Homo sapiens inositol 1,3,4-triphosphate 5/6 kinase (ITPK1), mRNA. |
| IDH1 | NM_005896.2 | 0.65 | Homo sapiens isocitrate dehydrogenase 1 (NADP+), soluble (IDH1), mRNA. |
| PPP1R1A | NM_006741.2 | 0.65 | Homo sapiens protein phosphatase 1, regulatory (inhibitor) subunit 1A (PPP1R1A), mRNA. |
| ITGB5 | NM_002213.3 | 0.64 | Homo sapiens integrin, beta 5 (ITGB5), mRNA. XM_944688 XM_944693 |
| LIPA | NM_000235.2 | 0.64 | Homo sapiens lipase A, lysosomal acid, cholesterol esterase (LIPA), transcript variant 2, mRNA. |
| C6orf145 | NM_183373.2 | 0.64 | PREDICTED: Homo sapiens chromosome 6 open reading frame 145 (C6orf145), mRNA. |
| HSD11B1 | NM_181755.1 | 0.64 | Homo sapiens hydroxysteroid (11-beta) dehydrogenase 1 (HSD11B1), transcript variant 2, mRNA. |
| NPLOC4 | NM_017921.1 | 0.64 | Homo sapiens nuclear protein localization 4 homolog (S. cerevisiae) (NPLOC4), mRNA. |
| LOC92755 | XR_018705.2 | 0.64 | PREDICTED: Homo sapiens misc_RNA (LOC92755), miscRNA. |
| LGALS8 | NM_201545.1 | 0.64 | Homo sapiens lectin, galactoside-binding, soluble, 8 (LGALS8), transcript variant 4, mRNA. |
| GDE1 | NM_016641.3 | 0.63 | Homo sapiens glycerophosphodiester phosphodiesterase 1 (GDE1), mRNA. |
| KLC1 | NM_182923.2 | 0.63 | Homo sapiens kinesin light chain 1 (KLC1), transcript variant 1, mRNA. |
| PGD | NM_002631.2 | 0.63 | Homo sapiens phosphogluconate dehydrogenase (PGD), mRNA. |
| RARRES1 | NM_206963.1 | 0.63 | Homo sapiens retinoic acid receptor responder (tazarotene induced) 1 (RARRES1), transcript variant 1, mRNA. |
| CD74 | NM_001025158.1 | 0.63 | Homo sapiens CD74 molecule, major histocompatibility complex, class II invariant chain (CD74), transcript variant 1, mRNA. |
| PCK1 | NM_002591.2 | 0.63 | Homo sapiens phosphoenolpyruvate carboxykinase 1 (soluble) (PCK1), mRNA. |
| AADACL1 | NM_020792.3 | 0.62 | Homo sapiens arylacetamide deacetylase-like 1 (AADACL1), mRNA. |
| VAC14 | NM_018052.3 | 0.62 | Homo sapiens Vac14 homolog (S. cerevisiae) (VAC14), mRNA. |
| ELAC2 | NM_018127.4 | 0.62 | Homo sapiens elaC homolog 2 (E. coli) (ELAC2), mRNA. |
| CD52 | NM_001803.2 | 0.62 | Homo sapiens CD52 molecule (CD52), mRNA. |
| PLCD4 | NM_032726.2 | 0.62 | Homo sapiens phospholipase C, delta 4 (PLCD4), mRNA. |
| FMO3 | NM_006894.4 | 0.62 | Homo sapiens flavin containing monooxygenase 3 (FMO3), transcript variant 1, mRNA. |
| MED8 | NM_001001653.1 | 0.62 | Homo sapiens mediator of RNA polymerase II transcription, subunit 8 homolog (S. cerevisiae) (MED8), transcript variant 5, mRNA. |
| P8 | NM_012385.1 | 0.62 | Homo sapiens p8 protein (candidate of metastasis 1) (P8), mRNA. |
| MATK | NM_139354.2 | 0.62 | Homo sapiens megakaryocyte-associated tyrosine kinase (MATK), transcript variant 3, mRNA. |
| PAK1 | NM_002576.3 | 0.62 | Homo sapiens p21/Cdc42/Rac1-activated kinase 1 (STE20 homolog, yeast) (PAK1), mRNA. |
| ERGIC3 | NM_198398.1 | 0.62 | Homo sapiens ERGIC and golgi 3 (ERGIC3), transcript variant 1, mRNA. |
| ALDOC | NM_005165.2 | 0.62 | Homo sapiens aldolase C, fructose-bisphosphate (ALDOC), mRNA. |
| FADS3 | NM_021727.3 | 0.62 | Homo sapiens fatty acid desaturase 3 (FADS3), mRNA. |
| SH3GLB1 | NM_016009.2 | 0.61 | Homo sapiens SH3-domain GRB2-like endophilin B1 (SH3GLB1), mRNA. |
| CBR1 | NM_001757.2 | 0.61 | Homo sapiens carbonyl reductase 1 (CBR1), mRNA. |
| SLC6A10P | NM_198857.1 | 0.61 | Homo sapiens solute carrier family 6 (neurotransmitter transporter, creatine), member 10 (pseudogene) (SLC6A10P) on chromosome 16. |
| RRAS2 | NM_012250.3 | 0.61 | Homo sapiens related RAS viral (r-ras) oncogene homolog 2 (RRAS2), mRNA. |
| OLFM2 | NM_058164.1 | 0.61 | Homo sapiens olfactomedin 2 (OLFM2), mRNA. |
| TFRC | NM_003234.1 | 0.61 | Homo sapiens transferrin receptor (p90, CD71) (TFRC), mRNA. |
| CIDEC | NM_022094.2 | 0.61 | Homo sapiens cell death-inducing DFFA-like effector c (CIDEC), mRNA. |
| ACO2 | NM_001098.2 | 0.61 | Homo sapiens aconitase 2, mitochondrial (ACO2), nuclear gene encoding mitochondrial protein, mRNA. |
| CALB2 | NM_001740.2 | 0.61 | Homo sapiens calbindin 2 (CALB2), transcript variant CALB2c, mRNA. |
| TENC1 | NM_198316.1 | 0.61 | Homo sapiens tensin like C1 domain containing phosphatase (tensin 2) (TENC1), transcript variant 3, mRNA. |
| DYNLL2 | NM_080677.1 | 0.60 | Homo sapiens dynein, light chain, LC8-type 2 (DYNLL2), mRNA. |
| NMB | NM_021077.3 | 0.60 | Homo sapiens neuromedin B (NMB), transcript variant 1, mRNA. |
| CS | NM_004077.2 | 0.60 | Homo sapiens citrate synthase (CS), nuclear gene encoding mitochondrial protein, mRNA. |
| CPVL | NM_031311.2 | 0.60 | Homo sapiens carboxypeptidase, vitellogenic-like (CPVL), transcript variant 2, mRNA. |
| SPOCD1 | NM_144569.3 | 0.60 | Homo sapiens SPOC domain containing 1 (SPOCD1), mRNA. |
| MOCOS | NM_017947.1 | 0.60 | Homo sapiens molybdenum cofactor sulfurase (MOCOS), mRNA. |
| MAGED2 | NM_201222.1 | 0.60 | Homo sapiens melanoma antigen family D, 2 (MAGED2), transcript variant 3, mRNA. |
| MS4A7 | NM_206938.1 | 0.60 | Homo sapiens membrane-spanning 4-domains, subfamily A, member 7 (MS4A7), transcript variant 2, mRNA. |
| ALDH3B1 | NM_001030010.1 | 0.60 | Homo sapiens aldehyde dehydrogenase 3 family, member B1 (ALDH3B1), transcript variant 1, mRNA. |
| CD151 | NM_139030.2 | 0.60 | Homo sapiens CD151 molecule (Raph blood group) (CD151), transcript variant 5, mRNA. |
| RBPMS | NM_001008712.1 | 0.60 | Homo sapiens RNA binding protein with multiple splicing (RBPMS), transcript variant 3, mRNA. |
| RGS20 | NM_170587.1 | 0.60 | Homo sapiens regulator of G-protein signaling 20 (RGS20), transcript variant 1, mRNA. |
| LOC730908 | XM_001717941.1 | 0.60 | PREDICTED: Homo sapiens hypothetical LOC730908, transcript variant 2 (LOC730908), mRNA. |
| HSPB7 | NM_014424.3 | 0.59 | Homo sapiens heat shock 27kDa protein family, member 7 (cardiovascular) (HSPB7), mRNA. |
| LOC731486 | NM_198277.1 | 0.59 | PREDICTED: Homo sapiens hypothetical protein LOC731486 (LOC731486), mRNA. |
| CRYAB | NM_001885.1 | 0.59 | Homo sapiens crystallin, alpha B (CRYAB), mRNA. |
| RETSAT | NM_017750.2 | 0.59 | Homo sapiens retinol saturase (all-trans-retinol 13,14-reductase) (RETSAT), mRNA. |
| MEGF9 | NM_001080497.1 | 0.59 | Homo sapiens multiple EGF-like-domains 9 (MEGF9), mRNA. |
| NEU1 | NM_000434.2 | 0.59 | Homo sapiens sialidase 1 (lysosomal sialidase) (NEU1), mRNA. |
| DNASE2B | NM_021233.2 | 0.59 | Homo sapiens deoxyribonuclease II beta (DNASE2B), transcript variant 1, mRNA. |
| GRN | NM_002087.2 | 0.59 | Homo sapiens granulin (GRN), mRNA. |

**Supplementary Table S3:** *List of all differentially expressed genes in gluteal SAT in response to exercise training based on log2 fold change > 0.58 (up and down-regulated)*

| **SYMBOL** | **SEARCH_KEY** | **logFC** | **DEFINITION** |
| --- | --- | --- | --- |
| ***Up-regulated genes in gSAT after exercise training*** | | | |
| MMP9 | NM_004994.2 | -1.44 | Homo sapiens matrix metallopeptidase 9 (gelatinase B, 92kDa gelatinase, 92kDa type IV collagenase) (MMP9), mRNA. |
| SPP1 | NM_001040058.1 | -1.24 | Homo sapiens secreted phosphoprotein 1 (SPP1), transcript variant 1, mRNA. |
| SPP1 | NM_000582.2 | -1.20 | Homo sapiens secreted phosphoprotein 1 (SPP1), transcript variant 2, mRNA. |
| APOC1 | NM_001645.3 | -1.14 | Homo sapiens apolipoprotein C-I (APOC1), mRNA. |
| ITGAX | NM_000887.3 | -1.04 | Homo sapiens integrin, alpha X (complement component 3 receptor 4 subunit) (ITGAX), mRNA. |
| TM4SF19 | NM_138461.2 | -1.04 | Homo sapiens transmembrane 4 L six family member 19 (TM4SF19), mRNA. |
| IFI30 | NM_006332.3 | -1.02 | Homo sapiens interferon, gamma-inducible protein 30 (IFI30), mRNA. |
| PLA2G7 | NM_005084.2 | -0.94 | Homo sapiens phospholipase A2, group VII (platelet-activating factor acetylhydrolase, plasma) (PLA2G7), mRNA. |
| LAPTM5 | NM_006762.1 | -0.93 | Homo sapiens lysosomal multispanning membrane protein 5 (LAPTM5), mRNA. |
| CISH | NM_145071.1 | -0.89 | Homo sapiens cytokine inducible SH2-containing protein (CISH), mRNA. |
| TM4SF19 | NM_138461.1 | -0.85 | PREDICTED: Homo sapiens transmembrane 4 L six family member 19, transcript variant 2 (TM4SF19), mRNA. |
| DHRS9 | NM_005771.3 | -0.84 | Homo sapiens dehydrogenase/reductase (SDR family) member 9 (DHRS9), transcript variant 1, mRNA. |
| COL1A1 | NM_000088.2 | -0.83 | Homo sapiens collagen, type I, alpha 1 (COL1A1), mRNA. |
| FCER1G | NM_004106.1 | -0.82 | Homo sapiens Fc fragment of IgE, high affinity I, receptor for; gamma polypeptide (FCER1G), mRNA. |
| CCL22 | NM_002990.3 | -0.80 | Homo sapiens chemokine (C-C motif) ligand 22 (CCL22), mRNA. |
| FCGBP | NM_003890.1 | -0.79 | Homo sapiens Fc fragment of IgG binding protein (FCGBP), mRNA. |
| APOE | NM_000041.2 | -0.79 | Homo sapiens apolipoprotein E (APOE), mRNA. |
| HP | NM_005143.2 | -0.78 | Homo sapiens haptoglobin (HP), mRNA. |
| CHI3L1 | NM_001276.2 | -0.77 | Homo sapiens chitinase 3-like 1 (cartilage glycoprotein-39) (CHI3L1), mRNA. |
| LOC653879 | XM_936226.1 | -0.77 | PREDICTED: Homo sapiens similar to Complement C3 precursor (LOC653879), mRNA. |
| SREBF1 | NM_004176.3 | -0.76 | Homo sapiens sterol regulatory element binding transcription factor 1 (SREBF1), transcript variant 2, mRNA. |
| MATK | NM_139354.2 | -0.74 | Homo sapiens megakaryocyte-associated tyrosine kinase (MATK), transcript variant 3, mRNA. |
| NNMT | NM_006169.2 | -0.74 | Homo sapiens nicotinamide N-methyltransferase (NNMT), mRNA. |
| CAPG | NM_001747.2 | -0.73 | Homo sapiens capping protein (actin filament), gelsolin-like (CAPG), mRNA. |
| ERGIC3 | NM_198398.1 | -0.73 | Homo sapiens ERGIC and golgi 3 (ERGIC3), transcript variant 1, mRNA. |
| F13A1 | NM_000129.2 | -0.72 | Homo sapiens coagulation factor XIII, A1 polypeptide (F13A1), mRNA. |
| ACP5 | NM_001611.2 | -0.72 | Homo sapiens acid phosphatase 5, tartrate resistant (ACP5), mRNA. |
| FAIM3 | NM_005449.3 | -0.71 | Homo sapiens Fas apoptotic inhibitory molecule 3 (FAIM3), mRNA. |
| PLTP | NM_006227.2 | -0.69 | Homo sapiens phospholipid transfer protein (PLTP), transcript variant 2, mRNA. |
| SLC43A3 | NM_017611.2 | -0.69 | Homo sapiens solute carrier family 43, member 3 (SLC43A3), mRNA. |
| LIPA | NM_000235.2 | -0.69 | Homo sapiens lipase A, lysosomal acid, cholesterol esterase (LIPA), transcript variant 2, mRNA. |
| GRN | NM_002087.2 | -0.68 | Homo sapiens granulin (GRN), mRNA. |
| FCGBP | XM_940656.1 | -0.68 | PREDICTED: Homo sapiens Fc fragment of IgG binding protein (FCGBP), mRNA. |
| TM7SF4 | NM_030788.2 | -0.66 | Homo sapiens transmembrane 7 superfamily member 4 (TM7SF4), mRNA. |
| TYROBP | NM_003332.2 | -0.66 | Homo sapiens TYRO protein tyrosine kinase binding protein (TYROBP), transcript variant 1, mRNA. |
| FBP1 | NM_000507.2 | -0.66 | Homo sapiens fructose-1,6-bisphosphatase 1 (FBP1), mRNA. |
| COL6A2 | NM_058174.1 | -0.65 | Homo sapiens collagen, type VI, alpha 2 (COL6A2), transcript variant 2C2, mRNA. |
| NPL | NM_030769.1 | -0.64 | Homo sapiens N-acetylneuraminate pyruvate lyase (dihydrodipicolinate synthase) (NPL), mRNA. |
| RABGGTA | NM_182836.1 | -0.64 | Homo sapiens Rab geranylgeranyltransferase, alpha subunit (RABGGTA), transcript variant 1, mRNA. |
| NPL | NM_030769.1 | -0.64 | Homo sapiens N-acetylneuraminate pyruvate lyase (dihydrodipicolinate synthase) (NPL), mRNA. |
| RNH1 | NM_203385.1 | -0.61 | Homo sapiens ribonuclease/angiogenin inhibitor 1 (RNH1), transcript variant 4, mRNA. |
| C3 | NM_000064.1 | -0.61 | Homo sapiens complement component 3 (C3), mRNA. |
| LGMN | NM_001008530.1 | -0.61 | Homo sapiens legumain (LGMN), transcript variant 2, mRNA. |
| MVP | NM_005115.3 | -0.61 | Homo sapiens major vault protein (MVP), transcript variant 2, mRNA. |
| CILP | NM_003613.2 | -0.60 | Homo sapiens cartilage intermediate layer protein, nucleotide pyrophosphohydrolase (CILP), mRNA. |
| CYP27A1 | NM_000784.2 | -0.60 | Homo sapiens cytochrome P450, family 27, subfamily A, polypeptide 1 (CYP27A1), nuclear gene encoding mitochondrial protein, mRNA. |
| GFPT2 | NM_005110.1 | -0.60 | Homo sapiens glutamine-fructose-6-phosphate transaminase 2 (GFPT2), mRNA. |
| LOC92755 | XR_018705.2 | -0.59 | PREDICTED: Homo sapiens misc_RNA (LOC92755), miscRNA. |
| CD163 | NM_203416.1 | -0.59 | Homo sapiens CD163 molecule (CD163), transcript variant 2, mRNA. |
| LOC646294 | XR_019565.2 | -0.59 | PREDICTED: Homo sapiens misc_RNA (LOC646294), miscRNA. |
| EMILIN2 | NM_032048.2 | -0.59 | Homo sapiens elastin microfibril interfacer 2 (EMILIN2), mRNA. |
| ALCAM | NM_001627.2 | -0.59 | Homo sapiens activated leukocyte cell adhesion molecule (ALCAM), mRNA. |
| LOC649679 | XM_945045.1 | -0.59 | PREDICTED: Homo sapiens similar to Tubulin beta-4q chain, transcript variant 2 (LOC649679), mRNA. |
| CLDN7 | NM_001307.3 | -0.59 | Homo sapiens claudin 7 (CLDN7), mRNA. |
| ***Down-regulated genes in gSAT after exercise training*** | | | |
| LOC651309 | XM_942586.1 | 0.64 | PREDICTED: Homo sapiens hypothetical protein LOC651309 (LOC651309), mRNA. |
| PCDH9 | NM_020403.3 | 0.64 | Homo sapiens protocadherin 9 (PCDH9), transcript variant 1, mRNA. |
| NTM | NM_016522.2 | 0.63 | Homo sapiens neurotrimin (NTM), transcript variant 2, mRNA. |
| SLIT2 | NM_004787.1 | 0.62 | Homo sapiens slit homolog 2 (Drosophila) (SLIT2), mRNA. |
| NUTF2 | NM_005796.1 | 0.61 | Homo sapiens nuclear transport factor 2 (NUTF2), mRNA. |
|  | Hs.99472 | 0.60 | Homo sapiens mRNA; cDNA DKFZp564O0862 (from clone DKFZp564O0862) |
| FAM13A | NM_014883.2 | 0.59 | Homo sapiens family with sequence similarity 13, member A (FAM13A), transcript variant 1, mRNA. |

**Supplementary Table S4:** *List of all differentially expressed genes in abdominal SAT in response to exercise training based on log2 fold change > 0.58 (up and down-regulated)*

| **SYMBOL** | **SEARCH_KEY** | **logFC** | **DEFINITION** |
| --- | --- | --- | --- |
| ***Up-regulated genes in aSAT after exercise training*** | | | |
| ACTA1 | NM_001100.3 | -1.59 | Homo sapiens actin, alpha 1, skeletal muscle (ACTA1), mRNA. |
| FOLR3 | NM_000804.2 | -1.23 | Homo sapiens folate receptor 3 (gamma) (FOLR3), mRNA. |
| MYL2 | NM_000432.2 | -1.15 | Homo sapiens myosin, light chain 2, regulatory, cardiac, slow (MYL2), mRNA. |
| CHI3L2 | NM_004000.2 | -1.13 | Homo sapiens chitinase 3-like 2 (CHI3L2), transcript variant 1, mRNA. |
| MYH7 | NM_000257.1 | -1.09 | Homo sapiens myosin, heavy chain 7, cardiac muscle, beta (MYH7), mRNA. |
| COL1A1 | NM_000088.2 | -1.02 | Homo sapiens collagen, type I, alpha 1 (COL1A1), mRNA. |
| FLNC | NM_001458.2 | -0.98 | Homo sapiens filamin C, gamma (actin binding protein 280) (FLNC), mRNA. |
| CKM | NM_001824.2 | -0.96 | Homo sapiens creatine kinase, muscle (CKM), mRNA. |
| LTB | NM_002341.1 | -0.93 | Homo sapiens lymphotoxin beta (TNF superfamily, member 3) (LTB), transcript variant 1, mRNA. |
| FNDC1 | NM_032532.1 | -0.90 | Homo sapiens fibronectin type III domain containing 1 (FNDC1), mRNA. |
| CFB | NM_001710.4 | -0.89 | Homo sapiens complement factor B (CFB), mRNA. |
| HOPX | NM_139212.2 | -0.88 | Homo sapiens HOP homeobox (HOPX), transcript variant 3, mRNA. |
| SLN | NM_003063.1 | -0.84 | Homo sapiens sarcolipin (SLN), mRNA. |
| CXCL12 | NM_000609.4 | -0.82 | Homo sapiens chemokine (C-X-C motif) ligand 12 (stromal cell-derived factor 1) (CXCL12), transcript variant 2, mRNA. |
| ALPL | NM_000478.2 | -0.82 | Homo sapiens alkaline phosphatase, liver/bone/kidney (ALPL), transcript variant 1, mRNA. |
| MB | NM_005368.2 | -0.81 | Homo sapiens myoglobin (MB), transcript variant 1, mRNA. |
| MYBPC1 | NM_002465.2 | -0.80 | Homo sapiens myosin binding protein C, slow type (MYBPC1), transcript variant 2, mRNA. |
| PTGDS | NM_000954.5 | -0.80 | Homo sapiens prostaglandin D2 synthase 21kDa (brain) (PTGDS), mRNA. |
| C1QTNF9 | NM_178540.3 | -0.79 | Homo sapiens C1q and tumor necrosis factor related protein 9 (C1QTNF9), mRNA. |
| CXCL12 | NM_001033886.1 | -0.79 | Homo sapiens chemokine (C-X-C motif) ligand 12 (stromal cell-derived factor 1) (CXCL12), transcript variant 2, mRNA. |
| CPXM1 | NM_019609.3 | -0.75 | Homo sapiens carboxypeptidase X (M14 family), member 1 (CPXM1), mRNA. |
| IGDCC4 | NM_020962.1 | -0.74 | Homo sapiens immunoglobulin superfamily, DCC subclass, member 4 (IGDCC4), mRNA. |
| BASP1 | NM_006317.3 | -0.71 | Homo sapiens brain abundant, membrane attached signal protein 1 (BASP1), mRNA. |
| FNDC1 | NM_032532.1 | -0.69 | Homo sapiens fibronectin type III domain containing 1 (FNDC1), mRNA. |
| SMOC2 | NM_022138.1 | -0.69 | Homo sapiens SPARC related modular calcium binding 2 (SMOC2), mRNA. |
| ISLR | NM_005545.3 | -0.69 | Homo sapiens immunoglobulin superfamily containing leucine-rich repeat (ISLR), transcript variant 1, mRNA. |
| IL7R | NM_002185.2 | -0.68 | Homo sapiens interleukin 7 receptor (IL7R), mRNA. |
| IL7R | XM_937367.1 | -0.68 | PREDICTED: Homo sapiens interleukin 7 receptor (IL7R), mRNA. |
| PDPN | NM_001006625.1 | -0.67 | Homo sapiens podoplanin (PDPN), transcript variant 4, mRNA. |
| HP | NM_005143.2 | -0.67 | Homo sapiens haptoglobin (HP), mRNA. |
| GNLY | NM_012483.1 | -0.66 | Homo sapiens granulysin (GNLY), transcript variant 519, mRNA. |
| ATP8B2 | NM_020452.2 | -0.66 | Homo sapiens ATPase, class I, type 8B, member 2 (ATP8B2), transcript variant 1, mRNA. |
| CTSG | NM_001911.2 | -0.66 | Homo sapiens cathepsin G (CTSG), mRNA. |
| HRC | NM_002152.2 | -0.64 | Homo sapiens histidine rich calcium binding protein (HRC), mRNA. |
| FAM129A | NM_022083.1 | -0.64 | Homo sapiens family with sequence similarity 129, member A (FAM129A), transcript variant 2, mRNA. |
| F10 | NM_000504.2 | -0.64 | Homo sapiens coagulation factor X (F10), mRNA. |
| APCDD1L | NM_153360.1 | -0.64 | Homo sapiens adenomatosis polyposis coli down-regulated 1-like (APCDD1L), mRNA. |
| GZMA | NM_006144.2 | -0.63 | Homo sapiens granzyme A (granzyme 1, cytotoxic T-lymphocyte-associated serine esterase 3) (GZMA), mRNA. |
| C7 | NM_000587.2 | -0.63 | Homo sapiens complement component 7 (C7), mRNA. |
| CD3D | NM_001040651.1 | -0.63 | Homo sapiens CD3d molecule, delta (CD3-TCR complex) (CD3D), transcript variant 2, mRNA. |
| TCAP | NM_003673.2 | -0.63 | Homo sapiens titin-cap (telethonin) (TCAP), mRNA. |
| TMEM154 | NM_152680.1 | -0.62 | Homo sapiens transmembrane protein 154 (TMEM154), mRNA. |
| KBTBD10 | NM_006063.2 | -0.62 | Homo sapiens kelch repeat and BTB (POZ) domain containing 10 (KBTBD10), mRNA. |
| CHST15 | NM_015892.2 | -0.61 | Homo sapiens carbohydrate (N-acetylgalactosamine 4-sulfate 6-O) sulfotransferase 15 (CHST15), mRNA. |
| CD48 | NM_001778.2 | -0.61 | Homo sapiens CD48 molecule (CD48), mRNA. |
| KLRB1 | NM_002258.2 | -0.61 | Homo sapiens killer cell lectin-like receptor subfamily B, member 1 (KLRB1), mRNA. |
| MYOZ1 | NM_021245.2 | -0.61 | Homo sapiens myozenin 1 (MYOZ1), mRNA. |
| GALNTL1 | NM_020692.1 | -0.61 | Homo sapiens UDP-N-acetyl-alpha-D-galactosamine:polypeptide N-acetylgalactosaminyltransferase-like 1 (GALNTL1), mRNA. |
| LTB | NM_002341.1 | -0.60 | Homo sapiens lymphotoxin beta (TNF superfamily, member 3) (LTB), transcript variant 1, mRNA. |
| C1orf54 | NM_024579.1 | -0.60 | Homo sapiens chromosome 1 open reading frame 54 (C1orf54), mRNA. |
| CILP | NM_003613.2 | -0.59 | Homo sapiens cartilage intermediate layer protein, nucleotide pyrophosphohydrolase (CILP), mRNA. |
| LILRB3 | NM_006864.1 | -0.59 | Homo sapiens leukocyte immunoglobulin-like receptor, subfamily B (with TM and ITIM domains), member 3 (LILRB3), transcript variant 2, mRNA. |
| SEMA4D | NM_006378.2 | -0.59 | Homo sapiens sema domain, immunoglobulin domain (Ig), transmembrane domain (TM) and short cytoplasmic domain, (semaphorin) 4D (SEMA4D), mRNA. |
| TPSAB1 | NM_003294.3 | -0.59 | Homo sapiens tryptase alpha/beta 1 (TPSAB1), mRNA. |
| CD79A | NM_001783.2 | -0.59 | Homo sapiens CD79a molecule, immunoglobulin-associated alpha (CD79A), transcript variant 2, mRNA. |
| ***Down-regulated genes in aSAT after exercise training*** | | | |
| FOS | NM_005252.2 | 1.05 | Homo sapiens v-fos FBJ murine osteosarcoma viral oncogene homolog (FOS), mRNA. |
| FOSB | NM_006732.1 | 0.89 | Homo sapiens FBJ murine osteosarcoma viral oncogene homolog B (FOSB), mRNA. |
| CSN1S1 | NM_001025104.1 | 0.87 | Homo sapiens casein alpha s1 (CSN1S1), transcript variant 1, mRNA. |
| MYOC | NM_000261.1 | 0.82 | Homo sapiens myocilin, trabecular meshwork inducible glucocorticoid response (MYOC), mRNA. |
| TWIST1 | NM_000474.3 | 0.77 | Homo sapiens twist homolog 1 (Drosophila) (TWIST1), mRNA. |
| LOC643911 | XR_042101.1 | 0.76 | PREDICTED: Homo sapiens hCG1815491 (LOC643911), miscRNA. |
| CSN1S1 | NM_001890.1 | 0.76 | Homo sapiens casein alpha s1 (CSN1S1), transcript variant 1, mRNA. |
| MYOC | NM_000261.1 | 0.75 | Homo sapiens myocilin, trabecular meshwork inducible glucocorticoid response (MYOC), mRNA. |
| THBS4 | NM_003248.3 | 0.75 | Homo sapiens thrombospondin 4 (THBS4), mRNA. |
| LOC643911 | XM_931911.1 | 0.72 | PREDICTED: Homo sapiens hypothetical LOC643911 (LOC643911), mRNA. |
| TSTD1 | NM_001113206.1 | 0.71 | Homo sapiens thiosulfate sulfurtransferase (rhodanese)-like domain containing 1 (TSTD1), transcript variant 2, mRNA. |
| GLDN | NM_181789.1 | 0.65 | Homo sapiens gliomedin (GLDN), mRNA. |
| TMEM100 | NM_018286.1 | 0.65 | Homo sapiens transmembrane protein 100 (TMEM100), transcript variant 2, mRNA. |
| IRX5 | NM_005853.4 | 0.64 | Homo sapiens iroquois homeobox protein 5 (IRX5), mRNA. |
| TNMD | NM_022144.1 | 0.64 | Homo sapiens tenomodulin (TNMD), mRNA. |
| SYN2 | NM_133625.2 | 0.63 | Homo sapiens synapsin II (SYN2), transcript variant IIa, mRNA. |
| IRX5 | NM_005853.4 | 0.63 | Homo sapiens iroquois homeobox 5 (IRX5), mRNA. |
| AHNAK | NM_024060.2 | 0.61 | Homo sapiens AHNAK nucleoprotein (AHNAK), transcript variant 2, mRNA. |
| AHNAK | NM_024060.2 | 0.60 | Homo sapiens AHNAK nucleoprotein (AHNAK), transcript variant 1, mRNA. |
| NPY5R | NM_006174.2 | 0.60 | Homo sapiens neuropeptide Y receptor Y5 (NPY5R), mRNA. |
| CALB2 | NM_001740.2 | 0.59 | Homo sapiens calbindin 2 (CALB2), transcript variant CALB2c, mRNA. |
| GRB14 | NM_004490.2 | 0.59 | Homo sapiens growth factor receptor-bound protein 14 (GRB14), mRNA. |
